# Pro-aggregant Tau impairs mossy fiber plasticity due to structural changes and Ca^++^ dysregulation

**DOI:** 10.1186/s40478-015-0193-3

**Published:** 2015-04-03

**Authors:** Jochen Martin Decker, Lars Krüger, Astrid Sydow, Shanting Zhao, Michael Frotscher, Eckhard Mandelkow, Eva-Maria Mandelkow

**Affiliations:** German Center for Neurodegenerative Diseases (DZNE), Ludwig-Erhard-Allee 2, 53175 Bonn, Germany; Caesar Research Center, Ludwig-Erhard-Allee 2, 53175 Bonn, Germany; Max-Planck-Institute for Metabolism Research, Hamburg Outstation, c/o DESY, Notkestrasse 85, 22607 Hamburg, Germany; University Medical Center Hamburg-Eppendorf, Center for Molecular Neurobiology Hamburg, Institute for Structural Neurobiology, Falkenried 94, 20251 Hamburg, Germany

**Keywords:** Calcium dysregulation, Long-term depression, Mossy fiber pathway, Presynapse, Tau

## Abstract

**Introduction:**

We used an inducible mouse model expressing the Tau repeat domain with the pro-aggregant mutation ΔK280 to analyze presynaptic Tau pathology in the hippocampus.

**Results:**

Expression of pro-aggregant Tau^RDΔ^ leads to phosphorylation, aggregation and missorting of Tau in area CA3. To test presynaptic pathophysiology we used electrophysiology in the mossy fiber tract. Synaptic transmission was severely disturbed in pro-aggregant Tau^RDΔ^ and Tau-knockout mice. Long-term depression of the mossy fiber tract failed in pro-aggregant Tau^RDΔ^ mice. We observed an increase in bouton size, but a decline in numbers and presynaptic markers. Both pre-and postsynaptic structural deficits are preventable by inhibition of Tau^RDΔ^ aggregation. Calcium imaging revealed progressive calcium dysregulation in boutons of pro-aggregant Tau^RDΔ^ mice. In N2a cells we observed this even in cells without tangle load, whilst in primary hippocampal neurons transient Tau^RDΔ^ expression alone caused similar Ca^++^ dysregulation. Ultrastructural analysis revealed a severe depletion of synaptic vesicles pool in accordance with synaptic transmission impairments.

**Conclusions:**

We conclude that oligomer formation by Tau^RDΔ^ causes pre- and postsynaptic structural deterioration and Ca^++^ dysregulation which leads to synaptic plasticity deficits.

**Electronic supplementary material:**

The online version of this article (doi:10.1186/s40478-015-0193-3) contains supplementary material, which is available to authorized users.

## **Introduction**

Several neurodegenerative disorders are characterized by pathological aggregation of the axonal protein Tau into “neurofibrillary tangles” classifying them as tauopathies [1]. The clinical signs of various tauopathies including Alzheimer disease (AD) and frontotemporal dementia with Parkinsonism linked to chromosome 17 (FTDP-17) correlate well with the anatomical localization of Tau aggregates in the brain [2]. Especially in AD, a correlation of hyperphosphorylated, aggregated Tau distribution and cognitive impairment is evident [3]. So far, most electrophysiological studies investigated Tau-related postsynaptic changes in area CA1 of the hippocampus [4-6]. However, it became increasingly obvious from animal models expressing mutant Tau that Tau pathology appears prominently in the stratum lucidum (s.l.) of area CA3 where axons of granule cells form the mossy fiber path [7-9]. In transgenic mice expressing the aggregation-prone human Tau repeat domain with the FTDP-17 mutation ΔK280 (termed pro-aggregant Tau^RDΔ^), exogenous mutant Tau co-aggregates with endogenous mouse Tau to assemble into neurofibrillary tangles in area CA3, whereas mice expressing anti-aggregant Tau (Tau_RD_ΔK280PP, termed Tau^RDΔPP^, containing two additional prolines inhibiting ß-structure) do not show pathology [8]. In Alzheimer disease pathological Tau is found within the hippocampal formation [10]. Recent observations on human AD brains though demonstrated abnormal Tau phosphorylation in thorny excrescences, the specialized spine structures characteristic for CA3 pyramidal neurons which constitute the postsynaptic target of mossy fiber boutons [11]. The density of thorny excrescences was decreased whereas their size was increased during the course of AD [12]. Moreover, immunoreactivity with AD-diagnostic antibodies PHF1, Alz50, and TG3 [13-15] were found in DG granule cells of AD patients, pointing to a clinical relevance of the mossy fiber pathway to Tau-related disease progression.

In hibernating ground squirrels, Tau becomes highly phosphorylated and missorted from its normal axonal localization into the somatodendritic compartment, which leads to the retraction of mossy fibers from CA3 postsynapses [16]. This process is reversible and suggests a physiological role of Tau in mossy fiber plasticity. In addition, Tau plays an important role in mossy fiber reorganization during development [17], and Tau is involved in the pathological sprouting of mossy fibers induced by kainate injection to generate epileptic seizures [18].

Thorny excrescences are innervated by “giant” mossy fiber boutons, which are characterized by their low basal transmitter release probability [19]. This is maintained by the action of presynaptic adenosine (A1) receptors [20] and group II metabotropic glutamate receptors (mGluRII) [21]. Long term depression (LTD) at the mossy fiber-CA3 synapse is expressed presynaptically as a reduction in neurotransmitter release [22] and depends on an activity-dependent rise in intracellular calcium (Ca^++^) concentrations [23].

In the present study we wanted to clarify to what extent pro-aggregant human Tau^RDΔ^ influences presynaptic plasticity in the hippocampus. For this purpose, we performed functional and morphological analyses of the mossy fiber synapse in Tau aggregation models and Tau knockout (TKO) mice. We found that aggregating Tau causes pre- and postsynaptic morphological changes at the mossy fiber-CA3 synapse and an impairment of LTD in pro-aggregant Tau^RDΔ^. Endogenous Tau was necessary for basal synaptic transmission at that synapse. In vitro Ca^++^ imaging studies with organotypic hippocampal slices, primary hippocampal neurons and N2a cells expressing pro-aggregant Tau^RDΔ^ demonstrated dysregulation of neuronal calcium influx as a possible basis for plasticity impairment. Structural synaptic changes could be prevented partially by the inhibition of Tau aggregation.

## **Materials and methods**

### Generation of Tau^RDΔ^ transgenic mice

Regulatable transgenic mice expressing the four-repeat domain of human Tau (Tau^RD^, residues 244–372) were generated as described previously [8]. The pro-aggregant form Tau^RDΔ^ carried the mutation ∆K280, the anti-aggregant form Tau^RDΔPP^ carried the mutations ∆K280/I277P/I308P where the additional prolines prevent aggregation through β-structure (for details see [7]). Transgene expression was monitored and quantified by bioluminescence of luciferin via the co-expressed luciferase (for details see [9,24]. The Tau knockout mouse was created by H. Dawson and colleagues by homologous recombination replacing exon 1 with a neomycin expression cassette [25]. Non-transgenic littermates of pro- and anti-aggregant mutants were used as controls. Transgenic animals were fed with doxycycline-containing pellets for 3 weeks to shut off mutant Tau gene expression in the first postnatal phase to prevent developmental disturbances. All animals were housed and tested according to standards of the German Animal Welfare Act.

### Hippocampal organotypic slice cultures

Hippocampal organotypic slice cultures were prepared following [26] with modifications [27]. Briefly, 8 days old mice were decapitated, brains were rapidly removed, and hippocampi dissected at 4°C. A McIIwain tissue chopper (Gabler, Bad Schwabach, Germany) was used to prepare 400 μm thick transverse slices which were transferred to semi porous cell culture inserts (Millipore, Bedford, MA, USA; 0.4 mm). Inserts containing 6 slices were placed in 6-well culture trays containing 1 mL of culture media (50% Minimum Essential Medium (MEM), 25% Hank’s Balanced Salt Solution (HBSS), penicillin/streptomycin [all from PAA, Pasching, Austria], 25% horse serum, 4.5 mg/mL glucose [Sigma-Aldrich, St. Louis, MO, USA], pH 7.4). The culture medium was changed on the first day after preparation and afterward every third day.

### Primary hippocampal cell culture

Primary hippocampal neurons were isolated from embryonic E18 Sprague Dawley rats and plated on poly-D-lysine-coated glass coverslips (50 g/ml) at a density of 50,000 cells per well in 24 well plates (Corning or Sarstedt) as described previously [28]. Neurons were transfected with Lipofectamin (Invitrogen) at DIV 14–23. Per coverslip 0.6 μg of plasmid DNA was used either with pAAV (plasmid Adeno Associated Virus)/pro-aggregant Tau^RDΔ^/IRES (Internal Ribosomal Entry Site)/hrGFP (human recombinant green fluorescent protein) or pAAV/IRES/hrGFP as a negative control plasmid. Transfections were carried out over a period of 72 h.

### Inducible N2a cell-line

Tau expressing N2a cell-lines were generated previously [29]. The stable N2a cell-lines N2a/Tet-ON/pro-aggregant Tau^RDΔ^ and N2a/Tet-ON/anti-aggregant Tau^RDΔPP^ were seeded on coverslips and grown confluently in Eagle’s minimum essential medium supplemented with 10% fetal calf serum, 2 mM glutamine, and 0.1% nonessential amino acids. The expression of constructs was induced by doxycycline and expression was continued for 4 days. On the fourth day expression was checked by bioluminescence. D-luciferin (Caliper Life Science) was added to the medium and bioluminescence was measured using an Ivis Spectrum imaging system (Caliper Life Science).

### Electron microscopy

For electron microscopic analysis of mossy fiber boutons, 4 adult wild-type animals and 4 pro-aggregant Tau^RDΔ^ transgenic mice were used. The animals were deeply anesthetized with a lethal dose of sodium pentobarbital and then transcardially perfused, first with 5 ml of physiological saline, followed by approximately 150 ml fixative solution (1% glutaraldehyde and 4% paraformaldehyde in 0.1 M phosphate buffer, pH 7.4). The fixative solution was perfused for approximately 15 min. Then, the brains were removed from the skulls and post-fixed in fixative solution over night at 4°C. After rinsing in 0.1 M phosphate buffer (pH 7.4), the brains were sectioned on a vibratome (Leica VT1000S) in the coronal plane at a thickness of 200 μm. Sections containing the dorsal (septal) hippocampus were processed for electron microscopy by placing the tissue in 1% OsO_4_ for 30 min. After careful washing (5 times, 5 min each) in distilled water, the sections were dehydrated in increasing concentrations of ethanol (block-staining the sections in 0.5% uranyl acetate in 70% ethanol for 1 hour). Thereafter, the sections were soaked in propylene oxide and finally embedded in Durcupan ACM resin (Fluca) and hardened at 56 ° C for at least 24 hours. The CA3 region was cut out and remounted on blank Durcupan blocks for thin sectioning. Thin sections were cut using an Ultracut (Leica) and a diamond knife. Sections were mounted on copper grids and post-stained with lead citrate. Electron micrographs of the stratum lucidum were taken at a magnification of × 5000. Ten mossy fiber boutons from each animal were used for quantitative analysis. Bouton area, number of vesicles, and the length of synaptic contact zones were measured using SIS software (Münster, Germany). Student’s t-test was used for statistical analysis.

### Histology

Gallyas silver staining and immunohistochemistry - using antibodies: 12E8 (pSer262/pSer356, 1:2000, gift of Dr. P. Seubert, Elan, S. San Francisco), PHF1 (pSer396/pSer404, 1:50, gift of Dr. P. Davies, Albert-Einstein College, N.Y.), MC1 (conformational changes of tau, 1:10, Dr. P. Davies) - were performed on 5 μm paraffin sections according to published procedures [3,8].

### Ca^++^ imaging of mossy fiber boutons

To load hippocampal slice cultures with Oregon Green 488 BAPTA 1-AM (OGB-1 AM), we added 4 μl 20% Pluronic F-127 in DMSO (Invitrogen) and 36 μl DMSO to a 50 μg vial of OGB-1 AM (Molecular Probes). The solution was added to slice culture medium to a final concentration of 10 μM. Slice cultures were submerged and incubated for 60 min at 37°C. Slices were washed with slice culture medium and then transferred to HEPES-buffered saline (HBS; 130 mM NaCl, 5.4 mM KCl; 10 mM HEPES, 25 mM glucose, 1.8 mM CaCl2, 1 mM MgCl2; ph 7.4) and allowed to recover for 30 min prior to an experiment. Imaging was performed with a Zeiss LSM 700 laser-scanning confocal microscope equipped with a 20x1A objective (Zeiss, Jena, Germany). Light from an argon laser (488 nm) was used for excitation and a FITC/GFP filter set for emission. Ca^++^ transients at individual mossy fiber boutons were evoked by applying 572 mM potassium chloride (KCl). The relative Ca^++^ increment is calculated as ΔF/F0, where F0 is resting fluorescence intensity of OGB-1 after subtraction of background fluorescence, and ΔF is the fluorescence increment from F0.

### Calcium imaging in cell-culture

Prior to calcium imaging experiments N2a cell cultures were induced with doxycycline (1 μg) for 4 days. N2a cell-lines and primary hippocampal neurons were washed at least 3 times with HEPES buffered saline. Thereafter cells were loaded with 10 μM Fura 2-AM (Molecular Probes) for 30 minutes at 37°C and 5% CO_2_ in HEPES-buffered saline. The Fura2-AM solution was removed and cells were washed three or more times and left for a few minutes in the incubator to allow complete de-esterification of the Fura dye. After washing, cultures were transferred to a submerged imaging chamber of an Examiner A1 microscope (Zeiss, Jena, Germany). Fura-2 fluorescence was imaged at room temperature (RT) in HEPES-buffered saline, using a 40× water immersion objective (Zeiss). The emission of Fura-2-loaded cells was collected at 510 nm after excitation at 340 and 380 nm respectively with a Sutter DCIV shutter (Sutter Instrument Co, Navato, CA, USA). Images were taken at a rate of 1 Hz. For baseline, intracellular Ca^++^ levels were recorded for at least 30 seconds subsequently cells were stimulated with a high potassium solution (286 mM KCl) by manual application and recorded for another 90 seconds. In primary hippocampal cultures, neurons and astrocytes were distinguished on the basis of their morphology and delayed intracellular Ca^++^ peak (~30 sec after high KCl for neurons and ~90 sec for astrocytes).

### Electrophysiology

To obtain preserved mossy fiber tracts, acute, transverse hippocampal slices were prepared from 13(±1) month-old female mice as described before [30]. ACSF was adjusted by increased Ca^++^ concentrations (126 mM NaCl, 26 mM NaHCO_3_, 3 mM KCl, 2.5 mM CaCl_2_, 1.3 mM MgSO_4_, 1.25 mM NaH_2_PO_4_ and 10 mM glucose, saturated with 95% O_2_/5% CO_2_). After 1.5 h recovery period in a homemade interface chamber, slices were transferred to a submerged recording chamber at 30°C. The mossy fiber tract was visualized by infrared (IR)/differential interference contrast (DIC) microscopy. As stimulation electrode, a patch-clamp pipette was used (1–2 MΩ) to stimulate mossy fibers (mf) in the region of the hilus. Recording electrodes (2.5-3 MΩ) were filled with ACSF and separated constantly 200 μm from stimulation electrode. fEPSPs were 10 × preamplified (custom made preamplifier) filtered at 1 kHz and sampled at 10 kHz using a HEKA double patch-clamp EPC 10 USB amplifier (HEKA Elektronik Dr. Schulze GmbH).

### Stimulation protocols and recording procedures

Constant current pulses were elicited every 20 s (0.05 Hz) with a pulse width of 0.1 ms. Input output (I/O) curves were generated using stimulus intensities ranging from 10–100 μA with an increment of 10 μA. In the same slice paired pulse facilitation (PPF) was evoked by a 50 ms inter-stimulus interval (ISI). In a next step frequency facilitation (ff) of the mossy fiber pathway was measured by switching stimulation frequency from 0.1 Hz to 1 Hz. Only slices which showed robust ff and PPF of approximately 1.5 were used further on for mf-LTD recordings. LTD was induced by 1 Hz stimulation for 15 min (900 pulses) at 50% of the maximum stimulus intensity as revealed initially during the I/O recording. At the end of each recording 5 μM of the mGluRII receptor antagonist DCGIV (Tocris Biosciences) diluted in ACSF was washed in for at least 5 min to test for mossy fiber specificity. In case the fEPSP slope and amplitude were not reduced to more than 50% of the initial value, slices were excluded from further mossy fiber analysis.

### Immunofluorescence

Brains from adult 13 month-old mice were fixed for 48 h in 4% PFA at 4°C and horizontal, hippocampal free-floating sections with a thickness of 80 μm were prepared with a vibratome (Leica VT 1200S, Wetzlar, Germany). Sections were rinsed with 0.4% TritonX in 5% horse serum for 1 h at RT. After washing with PBS first antibodies (PHF1 (pSer396/pSer404, Dr. P. Davies): 1:250; MC1 (Dr. P. Davies): 1:20) were incubated at 4°C over night. Secondary antibodies (donkey anti-mouse Alexa 488, Dianova) were incubated for 1:100 for 2 h at RT.

### Immunohistochemistry of slice cultures

Slice cultures were left attached on the Millicell membrane and stained as free-floating sections in 6-well plates. Cultures were first fixed with 4% paraformaldehyde (PFA) in phosphate buffered saline (PBS) (PAA, Austria) for 2 h at 4°C. After washing with PBS, slices were permeabilized by 0.1% TritonX-100/PBS for 90 min at RT. Slices were then blocked with 5% bovine serum albumin (BSA) for 2 h and afterwards incubated with primary antibody diluted in PBS for 3 days at 4°C. After washing three times with PBS, slices were incubated with secondary antibody for 2 days at 4°C. Finally, slices were incubated with the nuclear counterstain and dead cell indicator TO-PRO3 (Molecular Probes, US) for 15 min and washed three time with PBS before getting mounted with Permafluor mounting solution (Beckman Coulter, Paris, France. The following primary antibodies were used: pan-Tau antibody K9JA (Dako, Hamburg, Germany, Nr. A0024 (1:1000)), 12E8 (1:1000) for phosphorylated S262/S356 Tau (gift from Dr. P. Seubert, Elan Pharma, South San Francisco, CA, US) and Synaptophysin (1:1000; Sigma). All fluorescent (goat anti-rabbit/mouse cyanine 2 and 3)-labeled secondary antibodies were from Dianova (Hamburg, Germany) (1:1000).

### DiI labeling of organotypic slice cultures

Hippocampal slice cultures were fixed at day 10 in vitro (DIV 10) in 4% PFA for 2 hours, still attached to the semi-porous cell culture inserts. A DiI crystal was placed on the dentate gyrus followed by an incubation of 10 days at room temperature in 4% PFA to allow diffusion. Mossy fiber boutons and dendritic spines were imaged by confocal microscopy using tetramethyl rhodamine isothiocyanate (TRITC)-filter settings. Image J (NIH, USA) was used to measure morphological parameters such as spine density, mf bouton diameter and filopodia from resulting Z-stacks. For 3D surface determination, boutons were reconstructed by Imaris software (Version 7.6.4; Bitplane, Switzerland). For structural analysis at least 10 slices were prepared from 5 different animals per group.

### Drug application

Slice cultures were treated with compound bb14, a rhodanine-based Tau aggregation inhibitor identified in previous screens [31,32]. Compound bb14 was dissolved in 100% DMSO and added to the culture media at a final concentration of 15 μM (0.15% (v/v) DMSO). Control slices were treated with the same amount of DMSO alone. In all experiments treatment was done for the entire cultivation period starting at DIV 1. During treatment, the compound was replenished once a day after preparation and later on every 3rd day.

### Statistics

Throughout the study error bars denote standard error of the mean (SEM) except for ultrastructural analysis where errors denote standard deviation. Statistical hypothesis testing was done by using Prism5 (GraphPad, La Jolla, CA, USA), Statistica (Statsoft, Europe GmbH, Hamburg) and OriginPro8.5 (OriginLab cooperation, Northhampton, MA, USA). Evaluation of data was performed either by Student’s t-test or One way ANOVA followed by Tukey’s post-hoc test for morphological measurements in organotypic slices and multiple way repeated measure ANOVA was used for comparisons in electrophysiological recordings. Moreover, when groups did not have normal distribution the paired sample Wilcoxon signed rank test was used as a nonparametric test. p-values are indicated as follows: * p < 0.05, ** p < 0.01, and ***p < 0.001. Additional file 1: Supplemental experimental procedures.

## **Results**

### Endogenous Tau becomes phosphorylated and aggregated in area CA3 of the hippocampus due to expression of the human Tau repeat domain ΔK280

**Figure 1:**
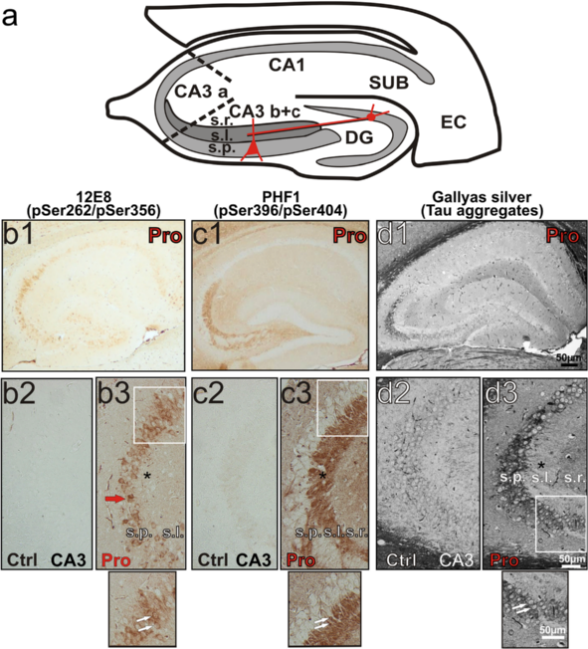
**Tau pathology in** ***stratum pyramidale*** **and** ***stratum lucidum*** **of area CA3 in sagittal sections from 13 ± 1 month old pro-aggregant Tau** ^**RDΔ**^ **mice. (a)** Schematic drawing of a hippocampal mouse section containing the entorhinal cortex (EC). The drawing depicts the main hippocampal output region, the subiculum (SUB) followed by the cornu ammonis (CA) regions with areas CA1, CA3a, CA3b, and CA3c (hilus). The main input region from the EC to the hippocampus is the dentate gyrus (DG) containing the granule cells (red) which project to proximal apical dendrites of area CA3 principal cells (red). s.r. = *stratum radiatum*; s.l. = *stratum lucidum*; s.p. = *stratum pyramidale*. **(b1-b3)** Somatodendritic mislocalization of phosphorylated Tau (12E8 immunoreactivity) in pyramidal neurons (B3 red arrow) and 12E8-immunoreactivity of *stratum lucidum* (s.l.,b3, asterisk) of the CA3 region in 13 ± 1 month-old pro-aggregant mice (Pro). In contrast there is no 12E8 immunoreactivity in area CA3 of control littermates (Ctrl, b2). Magnification square from region (**b3**, white square) depicts 12E8 immunoreactivity in somata of pyramidal neurons (white arrows). **(c1-c3)** PHF1-Tau antibody detects Tau phosphorylation exclusively in *stratum lucidum* of pro-aggregant mice (Pro, c1). CA3 pyramidal cell bodies and their dendrites are spared (**c3**, white arrows). Control littermates show no PHF1 immunoreactivity **(c2)**. A zoom-in of a region in **C3** (white square) demonstrates that PHF-1immunoreactivity is found in s.l. with apical dendrites of CA3 pyramidal neurons are spared (white arrows). **(d1-d3)** Using Gallyas silver staining to monitor Tau aggregation we found aggregates predominantly in CA3a-c pyramidal neurons of s.p. **(d1 and d3)**, whereas aggregation is absent in control littermates **(d2)**. Representative images from at least three different mice per group showing consistent staining were selected.

The “mossy fiber pathway” denotes axonal projections from dentate gyrus (DG) granule cells to area CA3 of the hippocampus, where “giant” boutons innervate thorny excrescences at CA3 pyramidal cell dendrites and interneurons (feed forward inhibition). These projections lying within the hippocampal layer *stratum lucidum* (s.l., Figure 1a) are specifically prone to pathological Tau hyperphosphorylation and aggregation in a FTDP-17 based mouse model overexpressing the repeat domain of Tau with the mutation ΔK280 (Tau^RDΔ^) in the forebrain [7,8].

Since Tau is known to be hyperphosphorylated in several tauopathies [33,34] we looked for Tau’s phospho-status by using different phosphorylation-dependent antibodies (for a complete list of antibodies see Additional file 2: Table S1). Using the phospho-dependent antibody 12E8 (pSer262/pSer356) we detected abnormally phosphorylated and mislocalized Tau in area CA3 a-c and DG in sagittal hippocampal sections from 13 ± 1 month-old mice (Figure 1b1). 12E8 immunoreactivity was detected in cell somata in the *stratum pyramidale* (s.p., Figure 1b1 and b3) but not in control littermate slices (Figure 1b2). We also used the phospho-dependent antibody PHF1 (pSer396/pSer404), a marker of pathological phosphorylation of endogenous Tau. PHF1 showed exclusively axonal immunoreactivity of mossy fibers in the *stratum lucidum* (Figure 1c1, Additional file 3: Figure S1b and e) but not in control littermates (Figure 1c2, Additional file 3: Figure S1a). Indeed detailed inspection of PHF1 immunoreactivity in area CA3 demonstrated that apical dendrites of pyramidal cells were not PHF1 positive (Figure 1c3, white arrows). We confirmed the presence of pathologically, phosphorylated endogenous Tau in the mossy fiber tract with the antibodies PHF1 and AT-8 (pSer202/Thr205; Additional file 3: Figure S1a-d) by immunofluorescence. Moreover we used immunofluorescence to trace single mossy fiber axons with PHF-1 immunoreactivity projecting from granule cells towards CA3 pyramidal cells (Additional file 3: Figure S1e). Finally, human Tau expression was confirmed by western blotting from CA3 hippocampal homogenates in pro-and anti-aggregant mice (Additional file 4: Figure S2a). The phosphorylation sites AT180 and AT8 were detected by western blotting in pro- and anti-aggregant mice but not in control littermates and Tau knockout mice (Additional file 4: Figure S2b-c) and we found weak immunoreactivity against the MC1 epitope demonstrating conformational change of Tau in that region (Additional file 4: Figure S2d).

Finally, Gallyas silver staining was applied to monitor Tau aggregation in our model (Figure 1d1). Similar to 12E8 immunoreactivity, Tau pathology occurred predominantly in somata of CA3 a-c pyramidal neurons of *stratum pyramidale* (Figure 1d1 and 1d3, white arrows), whereas there was no aggregation in control littermates (Figure 1d2).

### Tau is missorted to dendrites and somata in area CA3 of pro-aggregant Tau^RDΔ^ mice

The above observations prompted us to investigate the mossy fiber pathway in more detail. We stained horizontal hippocampal slices with the pan-Tau antibody K9JA. In 13 month-old control littermate mice, Tau was restricted to axons in mossy fiber bundles in s.l. and there was neither dendritic nor somatic Tau immunoreactivity throughout CA3 a-c (Additional file 5: Figure S3a). By contrast, in pro-aggregant Tau^RDΔ^ mice we observed missorting of Tau (endogenous and transgenic) into somato-dendritic compartments (Additional file 5: Figure S3b). Under these circumstances Tau was detectable in all three hippocampal layers (*s.r., s.l. and s.p.*, Additional file 5: Figure S3b, arrows). To better visualize mossy fibers and their projection targets (thorny excrescences) we used the lipophilic tracer DiI in combination with pan-Tau immunohistochemistry. This method highlighted Tau in mossy fiber boutons and postsynaptic, dendritic compartments in area CA3 a-c of pro-aggregant mice (Additional file 5: Figure S3f-h) whereas in control littermates pan-Tau immunoreactivity was consistently restricted to axonal and presynaptic “giant” bouton structures (Additional file 5: Figure S3c-e).

### Presynaptic pro-aggregant Tau^RDΔ^ impairs basal synaptic transmission, mossy fiber plasticity and reduces synaptic markers

**Figure 2:**
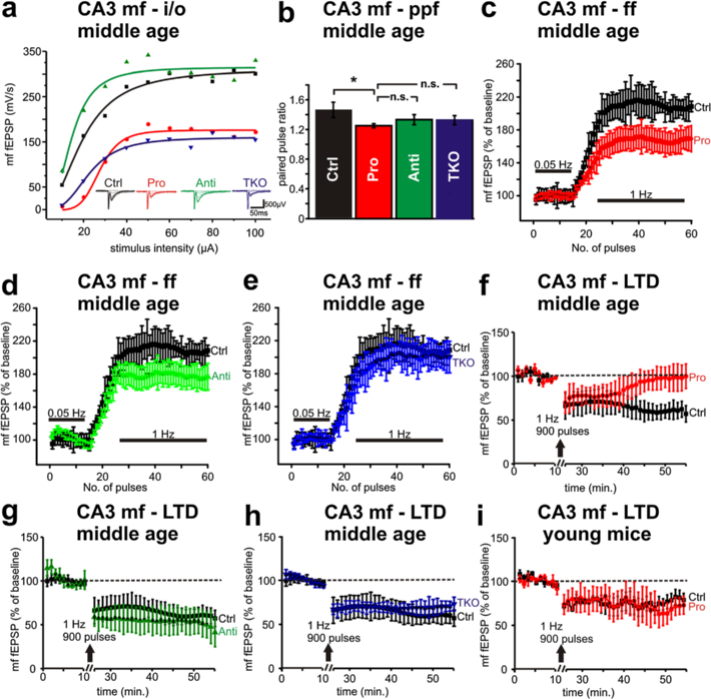
**Functional impairment of the mossy fiber pathway after 13 months of Tau** ^**RDΔ**^ **expression. (a)** Basal synaptic transmission of mossy fibers measured by input output curves (mf-i/o) is decreased in mossy fibers of pro-aggregant (Pro, red circles) and Tau knockout (TKO, blue triangles) mice compared to control littermates (Ctrl, black squares) and anti-aggregant (Anti; green triangles) animals. **(b)** Paired pulse facilitation of the mossy fiber pathway (mf-ppf) is significantly impaired in pro-aggregant mice (Pro, red column) compared to control littermates (Ctrl, white column), but not changed significantly if compared to anti-aggregant (Anti, green column) and Tau knockout (TKO, blue column) mice. **(c)** Frequency facilitation of the mossy fiber pathway (mf-ff) is reduced ~40% in pro-aggregant animals (Pro; red circles) compared to wildtype littermate control mice (Ctrl, black squares). **(d)** Anti-aggregant mice (Anti, green triangles) are less dramatically affected in terms of mf-ff impairment (~30%) compared to control mice. **(e)** Tau depleted mice (TKO, blue triangles) show no differences in mf-ff compared to control animals (Ctrl, black squares). **(f)** Long-term depression (LTD) of mossy fiber field excitatory postsynaptic potential (mf-LTD) induced by low frequency stimulation is not expressed in pro-aggregant mice (Pro, red circles), whereas control littermates (Ctrl, black squares) show a synaptic depression of about −30% of baseline. **(g)** Mossy fiber LTD in anti-aggregant mice (Anti, green triangles) is not different from control littermates. **(h)** Endogenous Tau is not necessary for normal mossy fiber LTD as shown by Tau knockout mice (TKO, blue triangles). **(i)** mf-LTD in 2 month old mice is expressed in pro-aggregant mice (Pro, red circles) and control littermates (Ctrl, black squares). Both groups show a synaptic depression of about −30% of baseline. Error bars represent SEM. Values represent recordings from at least 5 animals per group.

To test the patho-physiological consequences of presynaptic pro-aggregant Tau^RDΔ^, we used field potential recordings in *s.l.* of area CA3. We stimulated mossy fibers in the hilus region CA3 c, Figure 1a) and recorded mossy fiber field excitatory postsynaptic potentials (mf-fEPSP) in *stratum lucidum* of area CA3 b from 13 ± 1 month-old animals from pro-aggregant Tau^RDΔ^ and control littermates. We further included anti-aggregant Tau^RDΔPP^ [8] and Tau knockout (TKO) mice [25] in our study. Control littermates reached a maximum slope of mf-fEPSP on average at 312.9 mV/s (nonlinear curve fit (Hill); n = 4; Figure 2a), well comparable to the anti-aggregant mice average maximum slope (317.25 mV/s; nonlinear curve fit (Hill); n = 5; Figure 2a). In contrast pro-aggregant mice reached only a maximum mf-fEPSP slope of 177.34 mV/s (nonlinear curve fit (Hill); n = 5; Figure 2a). Notably TKO mice showed a maximum average mf-fEPSP of 161.2 mV/s (nonlinear curve fit (Hill); n = 6; Figure 2a). Pro-aggregant and remarkably TKO mice both revealed a pronounced decrease in the input/output (I/O) curve, compared with control littermates (multiple way repeated measure ANOVA: pro-aggregant vs. control littermates: group effect p = 0.014; TKO vs. control littermates: multiple way repeated measure ANOVA: group effect p = 0.001; Figure 2a). However, at the age of 2 months, the pro-aggregant mice did not differ from control littermates in basal synaptic transmission of the mossy fiber pathway demonstrating the progressive nature of this phenotype. Two month-old control littermate mice and pro-aggregant mice both reached the maximum fEPSP slope at 90 μA stimulation intensity (165.2 ± 22.4 mV/s, n = 6 and 169.1 ± 57.3 mV/s, n = 13 respectively; Figure 2a).

Next we investigated short-term plasticity measured by paired pulse facilitation (PPF). Pro-aggregant mice revealed a significant reduction of mf-PPF compared to control littermates (paired pulse ratio control: 1.46 ± 0.1, n = 24; paired pulse ratio pro-aggregant: 1.25 ± 0.03, n = 34; t-test: p = 0.023; Figure 2b), whereas anti-aggregant mice and Tau knockout mice differed not significantly from control littermates (anti-aggregant: 1.33 ± 0.06, n = 22, t-test: p = 0.296; TKO: 1.32 ± 0.06, n = 33, t-test: p = 0.222; Figure 2b). 2 month-old pro-aggregant mice however displayed no impairment of PPF compared to control littermates (paired pulse ratio control: 1.53 ± 0.12, n = 19 and paired pulse ratio pro-aggregant: 1.45 ± 0.14; n = 15; data not shown). Mossy fiber frequency facilitation (mf-ff) was decreased in pro-aggregant mice (last 20 stimulation pulses displayed in graphs: 167.4 ± 4% of baseline, n = 11 compared to control littermates: 208.4 ± 6% of baseline, n = 6; multiple way repeated measure ANOVA: group effect p = 0.019, Figure 2c). In anti-aggregant mice, mossy fiber frequency facilitation was scarcely and not significantly reduced confirming previous results (177.9 ± 5% of baseline, n = 4, Figure 2d; multiple way repeated measure ANOVA: group effect p = 0.455, [8]). TKO mice displayed frequency facilitation well comparable to control mice (199.3 ± 8% of baseline, n = 5, Figure 2e). Finally when comparing 2 month-old control littermates with pro-aggregant siblings we could not detect any differences in mf-ff (229.4 ± 7.7% of baseline, n = 10 and 240 ± 12.1% of baseline, n = 7; data not shown).

Mossy fiber long-term depression (mf-LTD) is expressed at the presynaptic site [22] and depends on proper neuronal Ca^++^ dynamics [23]. In control littermate mice, LTD induction by low frequency stimulation (LFS) led to an average synaptic depression of −31.1 ± 0.5% (n = 8, Figure 2f) of the initial baseline mf-fEPSP slope, whereas in pro-aggregant mice LTD was induced in the first 15 minutes after LFS, but was no longer present during the last 10 minutes of recording (reduction only −2.2 ± 0.4%; n = 7; Figure 2f). Therefore mf-LTD was significantly impaired in pro-aggregant mice compared to control littermates (paired sample Wilcoxon signed rank test, p = 0.0019, last 10 minutes of recording). In contrast, the same LTD protocol evoked a LTD expression closely comparable to control mice in anti-aggregant and TKO mice (Figure 2g and 2h respectively). Moreover comparing young cohorts of pro-aggregant mice and non-transgenic siblings we found no differences in LTD expression (last 10 minutes of recording, control littermates: −23.6 ± 1.2% of baseline recording and pro-aggregant: −31.8 ± 1.3% of baseline, Figure 2i). Consistently we found in CA3 homogenates dissected from pro-aggregant Tau^RDΔ^ mice a reduction of the presynaptic vesicle protein synapsin1 and synaptophysin (Additional file 3: Figure S2e and f). This was accompanied by a loss of the postsynaptic markers PSD95 and the NMDA receptor NR1 subunit (Additional file 3: Figure S2e and f).

### Pro-aggregant Tau^RDΔ^ leads to pathological Tau accumulation in presynapses and impairs synapse morphology

**Figure 3:**
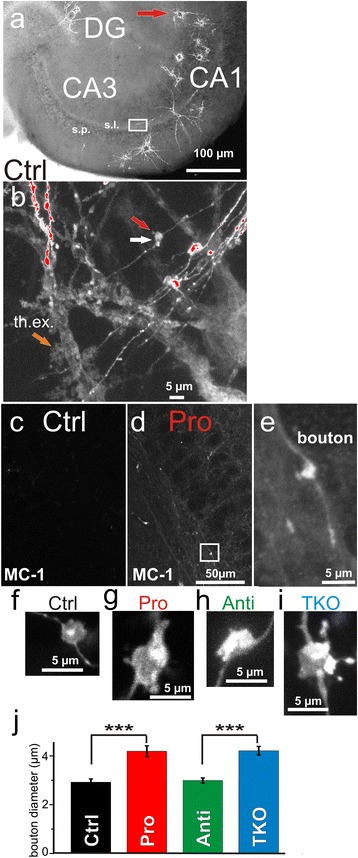
**Mossy fiber presynapse morphology is altered because of Tau** ^**RDΔ**^ **expression. (a)** Microphotograph depicting an example of an acute, horizontal slice preparation shot with DiI coated gold particles (red arrow) to obtain neuronal membrane staining. Hippocampal sub-regions dentate gyrus (DG), CA3 and CA1 are visible. **(b)** Higher magnification of region of interest marked in **(a)** (white square) located in *stratum lucidum* of area CA3. Characteristic structural, postsynaptic features of CA3 pyramidal neurons are highlighted (thorny excrescences (th.ex.), orange arrow). Moreover, mossy fiber axons with boutons and filopodia are visible and correspond to the criteria for bouton selection (red arrow = filopodium; white arrow = bouton body). **(c)** Immunohistological detection of conformation altered Tau with MC-1 antibody in a control littermate mouse at 13 ± 1 month, display no staining. **(d)** MC-1 immunofluorescence staining in aged matched pro-aggregant mouse (Pro) in s.l. Immunoreactivity is detected. **(e)** Higher magnification of the indicated region in (**d**; white square). MC-1 immunostaining revealed immunoreactivity in mossy fiber boutons. **(f)** Example of a mossy fiber “giant” bouton in control littermate mice (Ctrl). **(g)** Mossy fiber bouton of a pro-aggregant mouse (Pro) with filopodia emanating from the bouton surface. **(h)** Area CA3 mossy fiber bouton of an anti-aggregant mouse (Anti). **(i)** Example of a Tau knockout mouse bouton (TKO). **(j)** Measurement of bouton diameter in control littermate (black column), pro-aggregant (red column), anti-aggregant (green column) and Tau knockout mice (blue column) illustrating an increase of ~ 45% in diameter in pro-aggregant and Tau knockout mice. (10 slices prepared from at least 3 different animals per group). Error bars = SEM; p-value < 0.001***.

Mossy fiber pre-synapses, the “giant” boutons, have unique morphological features: Their diameter ranges between 2–5 μm and filopodia emanate from their surface [35-37]. We randomly labeled mossy fibers in acute, horizontal slices from 13 ± 1 month-old mice with the lipophilic dye DiI by using a biolistic gene-gun in order to identify mossy fiber “giant” boutons (Figure 3a). As morphological criteria for identification of “giant” boutons we defined proximity to thorny excrescences of CA3 pyramidal neurons, their size and at least 1 filopodium (Figure 3b). The diameter of such “giant” boutons (Figure 3f-j) showed a pronounced increase of ~45% in pro-aggregant Tau^RDΔ^ mice compared with control littermates (pro-aggregant: 4.2 ± 0.2 μm,

n = 25 and control: 2.8 ± 0.1 μm, n = 25, Two-sample t-test, p < 0.001; Figure 3f and g). In slices from anti-aggregant mice we did not find this pathological phenotype (bouton diameter: 2.99 ± 0.1 μm, n = 43; Figure 3h), whereas TKO mice displayed a size increase closely comparable to pro-aggregant mice (4.2 ± 0.2 μm, n = 33; Figure 3i). Enlarged boutons in pro-aggregant mice contain conformational altered endogenous Tau (detected with MC1 antibody; Figure 3d and e), which was not observed in control littermates (Figure 3c).

**Figure 4:**
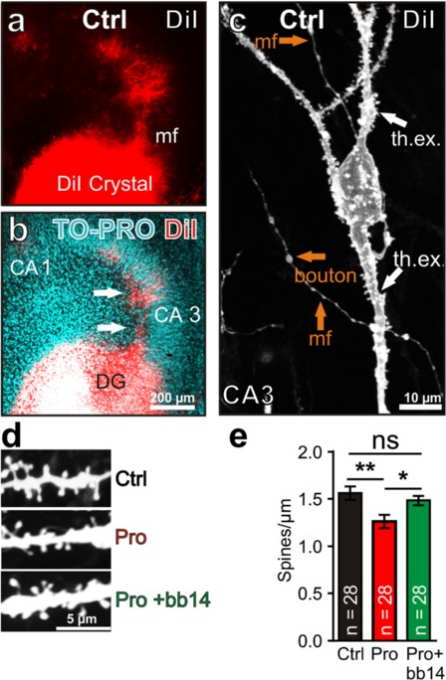
**Tau** ^**RDΔ**^ **expression causes morphological changes of the DG - CA3 postsynapse in hippocampal organotypic slice cultures at DIV 10. (a)** A DiI crystal was placed on the dentate gyrus of fixed hippocampal organotypic slices from pro-aggregant mice or control littermates. DiI labeled mossy fibers and CA3 pyramidal neurons were imaged 10 days later. **(b)** Nuclear counterstaining with TO-PRO was used to visualize hippocampal sub- regions. Arrows indicate DiI labeled mossy fibers. **(c)** Higher magnification of a single CA3 pyramidal neuron displays thorny excrescences (th.ex.) in the stratum lucidum and stratum oriens as well as projecting mossy fibers (mf) from the dentate gyrus granule cells with large boutons. **(d)** Examples of thorny excrescences dendritic spines from CA3 pyramidal neurons taken from control (Ctrl), pro-aggregant (Pro) and pro-aggregant slices treated with Tau aggregation inhibitor bb14 (Pro + bb14). **(e)** Dendritic spine density of CA3 neurons was reduced by ~20% in pro-aggregant (Pro) vs. control littermate slice cultures (Ctrl) (10 slices prepared from 5 different animals per group). This effect is abolished when pro-aggregant slice cultures are treated with the aggregation inhibitor bb14 (Pro + bb14) (One way ANOVA followed by Tukey’s post-hoc test ** p-value < 0.01; * p-value < 0.05). Error bars represent SEM.

**Figure 5:**
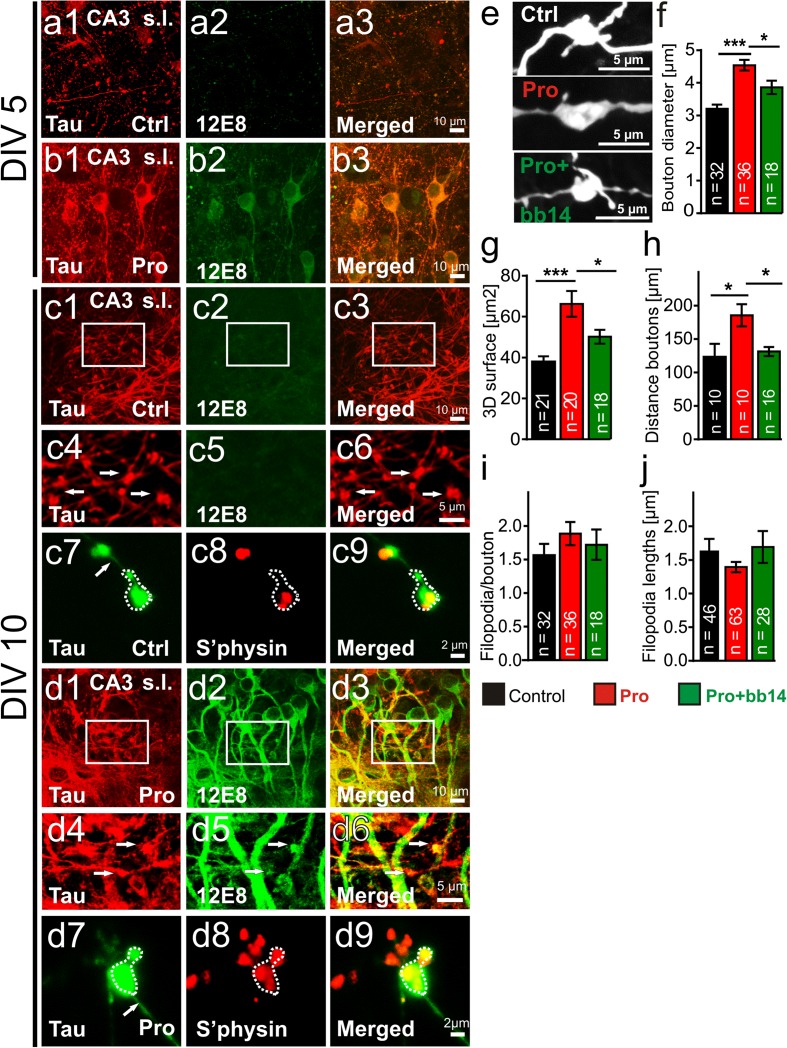
**Expression of Tau** ^**RDΔ**^ **causes morphological changes of mossy fiber boutons in hippocampal slice cultures.** Photomicrographs of slice cultures from control (Ctrl) or pro-aggregant-mice (Pro) at DIV5 (a1-b3) and DIV10 (c1-d9); area CA3-immunostaining against pan-Tau (K9JA, a1,b1,c1,d1), phospho-Tau (12E8, a2,b2,c2,d2) and merged (a3,b3,c3,d3) pictures are depicted. **(a1)** No 12E8-immunoreactivity is present in control slices at DIV5 (a1-a3) and DIV10 (c1-c3). **(b1)** In contrast, in pro-aggregant slices 12E8-immunoreactivity is observed in pyramidal cell somata and dendrites at DIV5 (b1-b3) and DIV10 (d1-d3). **(c4-c6)** Zoom-in images (from (c1)) showing mossy fiber boutons filled with Tau. No 12E8 phosphorylation was detected in control slices. (**d4**-**d6**, zoom-in from **d1**) In contrast, boutons in pro-aggregant slices contain phosphorylated Tau. **(c7-c9 + d7-d9)** Immunostaining against pan-Tau (green), synaptophysin (S’physin, red) and merged picture is shown in control **(c7-c9)** and pro-aggregant slices **(d7-d9)**. Arrows mark the axon shaft; boutons are marked by dotted line. **(e)** Higher magnification of single mossy fiber boutons (stained with DiI) in slices from control mice, pro-aggregant mice and pro-aggregant slices treated with compound bb14. **(f)** The bouton diameter was increased by ~42% in pro-aggregant slices in comparison to control and by only ~18% when compared to bb14-treated pro-aggregant slices. **(g)** The bouton surface was larger in pro-aggregant slices (by ~75%) vs. control and by only 32% vs. bb14-treated pro-aggregant slice cultures. **(h)** The average distance between boutons was increased by ~50% in pro-aggregant slices compared to control and bb14-treated pro-aggregant cultures. **(i)** Numbers of filopodia (control: 1.56; pro-aggregant: 1.89; pro-aggregant + bb14: 1.77) per bouton was only slightly affected in pro-aggregant slices. **(j)** The length of filopodia (control: 1.66 μm; pro-aggregant: 1.39 μm; pro-aggregant + bb14: 1.69 μm) was not changed in pro-aggregant slices. One way ANOVA followed by Tukey’s post-hoc test *** p- value < 0.001; * p-value < 0.05). Error bars represent SEM.

Next, we made use of organotypic slice cultures, since this system is particularly advantageous for long distance granule cell-CA3 axonal connections [38,39]. With DiI labeling we detected granule cell-CA3 mossy fiber connections in DIV 10 slices (Figure 4a and b), a time point when we already detected phosphorylated and mislocalized Tau in Tau^RDΔ^ slices (Figure 5d1-6), well comparable with results from acute slices. It was possible to label boutons as well as thorny excrescences in area CA3 (Figure 4c). In pro-aggregant slices the dendritic spine density (1.26 ± 0.07 spines/μm) was reduced by ~20% compared to control littermate slices (1.56 ± 0.07 spines/μm). This reduction was prevented by adding the Tau aggregation inhibitor bb14 [31] to pro-aggregant slices at DIV 0 (1.49 ± 0.05 spines/μm; F_(2/81)_ = 5.851; p = 0.0042; Figure 4d,e).

In organotypic slices we detected pan-Tau immunoreactivity in mossy fiber boutons from control littermates (Figure 5a1 and c1) and Tau^RDΔ^ (Figure 5b1 and d1) at DIV 5 and 10. We confirmed such Tau containing boutons by co-localization with presynaptic marker protein synaptophysin (Figure 5c7-c9 and d7-d9). In pro-aggregant Tau^RDΔ^ slices we observed strong 12E8 immunoreactivity in mf-boutons at DIV 10, which was not seen in control cultures either at DIV 5 or 10 (compare Figure 5d4-d6 with Figure 5a2 and c2). At DIV 5, 12E8 phosphorylation in Tau^RDΔ^ slices (Figure 5b1-b3) was weaker when compared to DIV 10 and in particular present in the postsynaptic compartment. In contrast, 12E8 immunoreactiviy was found in pre-and postsynaptic compartments at DIV 10 (Figure 5d4-d6).

The expression of pro-aggregant Tau^RDΔ^ led to a 42% increase in diameter of “giant” mf-boutons, comparable to hippocampal slices from adult mice, which was prevented when the aggregation inhibitor bb14 was added to the preparation (control: 3.20 ± 0.13 μm; pro-aggregant: 4.54 ± 0.16 μm; pro-aggregant + bb14: 3.86 ± 0.20 μm; F_(2/83)_ = 20.66; p < 0.0001; Figure 5e and f). In agreement with this, the 3D surface area of pro-aggregant boutons was larger (~74%) than in control littermate slices (control: 37.97 ± 2.69 μm^2^; pro-aggregant: 66.24 ± 6.30 μm^2^; pro-aggregant + bb14: 50.16 ± 3.41 μm^2^; F_(2/56)_ = 10.67; p < 0.0001; Figure 5g). We measured the average distance between mossy fiber boutons as a function of total excitation strength and observed an increased by ~51% in pro-aggregant Tau^RDΔ^ slices (185.4 ± 22.3 μm) compared to control littermates (122.9 ± 18.5 μm, Figure 5h). Again compound bb14 prevented this effect on mf-boutons (131.3 ± 6.7 μm, Figure 5f).

As a measure of feedforward inhibitory drive we determined the length and number of filopodia per bouton. Filopodia were defined as described elsewhere [40] as emanating from the main body of the bouton, with lengths of 1–5 μm. The filopodia number increased somewhat (~21%, control: 1.56 ± 0.17; pro-aggregant: 1.89 ± 0.17; pro-aggregant + bb14: 1.77 ± 0.23; F_(2/83)_ = 0.9031; p = 0.4092; Figure 5i) and correspondingly filopodia lengths were reduced to a similar extent so that the total filopodia length remained roughly the same (~17%, control: 1.66 ± 0.16 μm, pro-aggregant: 1.39 ± 0.08 μm, pro-aggregant + bb14: 1.69 ± 0.24 μm; F_(2/134)_ = 1.656, p = 0.2207; Figure 5j).

### The synaptic vesicle density from pro-aggregant mice was severely reduced in mossy fiber “giant” boutons

**Figure 6:**
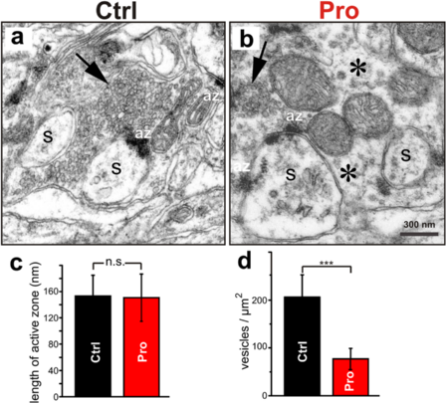
**Ultramicroscopy of mossy fiber boutons reveal severe synaptic vesicle reduction in mice expressing pro-aggregant Tau** ^**RD**Δ^ **. (a-b)** Electron micrographs of mossy fiber synapses from a control littermate **(a)** and pro-aggregant Tau^RDΔ^ transgenic mouse **(b)**. While the presynaptic mossy fiber bouton of the control animal is densely filled with clear synaptic vesicles (**a**, arrow), vesicle accumulations are rare in presynaptic mossy fiber boutons from pro-aggregant Tau^RDΔ^ mice (**b**, arrow). Rather, large parts of the presynaptic bouton area in the mutant are almost free of vesicles (asterisks). S = postsynaptic complex spines protruding into the presynaptic bouton. Scale bar: 300 nm. **(c)** Length of active zones and **(d)** number of synaptic vesicles/μm^2^ in mossy fiber synapses from control (Ctrl) and Tau^RDΔ^ transgenic animals. While there is no statistically significant difference in the length of active zones of mossy fiber synapses between control mice and mutants, the number of vesicles/μm^2^ bouton area is dramatically decreased in Tau^RDΔ^ transgenic mice. Data expressed as mean ± standard deviation. 4 animals per group ***p < 0.001; n.s. not significant; az = active zone.

Mossy fiber boutons in the stratum lucidum of CA3 were easily identified by their unique fine-structural characteristics. Thus, the thin unmyelinated preterminal mossy fiber axons gave rise to giant boutons that were densely filled with clear synaptic vesicles. Postsynaptic complex spines protruded deeply into the presynaptic boutons. At low magnification, no major differences in the fine structure of mossy fiber boutons were noticed between control littermate animals and pro-aggregant mice (13 ± 1 month, 4 mice per group). In fact, an estimation of the lengths of synaptic contact zones (active zones; az, Figure 6a and b) did not reveal statistically significant differences between genotypes (control: 153.2 ± 31.8 nm; pro-aggregant mice 150.7 ± 36.1 nm; p = 0.80876, Figure 6c). However, we regularly noticed a reduction in the number of synaptic vesicles in the mossy fiber boutons of pro-aggregant animals. While vesicles were densely packed and almost completely filled the mossy fiber terminals of control littermates (Figure 6a), vesicle accumulations were rare in the boutons from transgenic mice, and there were frequent bouton areas containing only a few scattered vesicles (Figure 6b). Indeed, the number of synaptic vesicles/μm^2^ bouton area was significantly decreased in Tau^RDΔ^ transgenic mice when compared to control littermates (control: 206.1 ± 47.0 vesicles/μm^2^; transgenic mice 77.3 ± 21.5 vesicles/μm^2^; p = 7.4487 ×10^−10^, Figure 6d).

### Ca^++^ influx is attenuated due to pro-aggregant Tau^RDΔ^ expression in mossy fiber boutons before tangle formation

**Figure 7:**
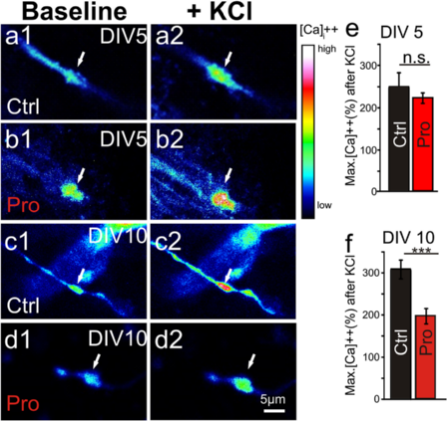
**Progressive impairment of Ca** ^**++**^ **regulation in mossy fiber boutons in slices of Tau** ^**RD**Δ^ **. (a1)** The photomicrograph shows a magnification of *stratum lucidum* in a hippocampal slice culture from a control mouse. The slice was labeled with the Ca^++^ indicator dye Oregon Green BAPTA-1-AM (OGB-1) at DIV 5. After labeling it was possible to locate “giant” mossy fiber boutons loaded with OGB (white arrow). **(a2)** After membrane depolarization with high KCl Ca^++^ is flowing into the bouton (white arrow). The increase of intracellular calcium is false-color coded as indicated on the right column. **(b1)** Analogue to **(a1-2)** a bouton (white arrow) in a pro-aggregant slice is depicted before and after **(b2)** membrane depolarization (white arrow). **(c1)** At DIV 10 a stronger Ca^++^ influx in boutons (white arrow) after membrane depolarization **(c2)** compared to boutons in DIV 5 slices **(a1-a2)** was observed. **(d1)** A strong decrease in Ca++ influx after depolarization **(d2)** is observed in boutons from pro-aggregant Tau^RDΔ^ slices compared to control slices at DIV 10. **(e)** Quantification of maximum intracellular Ca^++^ increase after high KCl application in mossy fiber boutons from control littermate slices (white column) and from pro-aggregant Tau^RDΔ^ slices (red column) at DIV 5. Note that the maximum intracellular Ca^++^ concentration did not change in comparison to boutons from control littermate DIV 5 slices. **(f)** Comparison of maximum intracellular Ca^++^ concentration in boutons from control littermate slices (white column) and from pro-aggregant Tau^RDΔ^ slices (red column) at DIV10. At DIV 10 the pro-aggregant Tau^RDΔ^ slices show a severe impairment in Ca^++^ influx after membrane depolarization. Note that the maximum intracellular Ca^++^ peak in control boutons increases with maturation of slices (compare e and f control). Error bars represent SEM. *** p-value < 0.001.

Normal Ca^++^ signaling during resting and active neuronal states is essential for neuronal survival and synaptic plasticity [41]. In the present study we wanted to know if depolarization dependent Ca^++^ dysregulation at the mossy fiber presynapse could be responsible for the described effects of pro-aggregant Tau^RDΔ^ on transmission and plasticity. Therefore we imaged Ca^++^ influx in mossy fiber boutons in *s.l.* of area CA3 in slice cultures loaded with Oregon green bapta (OGB, Figure 7). After membrane depolarization, we observed an increase of intracellular Ca^++^ concentration in axons with a peak in bouton-like structures compared with Ca^++^ influx at the axonal shaft (data not shown), indicating that functional ion channels are enriched on the bouton membrane. At DIV 5 the KCl-induced Ca ^++^ influx was comparable in boutons from pro-aggregant Tau^RDΔ^ slices and control littermates (pro-aggregant: 222.1 ± 12.4%, n = 10 and control: 249.7 ± 32.8% of baseline, n = 9; prepared from at least 5 animals per group, Figure 7a-b and e). However at a later time point (DIV 10) there was a pronounced reduction by 106.4% in pro-aggregant Tau^RDΔ^ expressing slices, compared with controls (control: 302.3 ± 21.1% of baseline, n = 20 and pro-aggregant: 195.9 ± 18.2% of baseline, n = 17; prepared from at least 5 animals per group) Figure 7c-d and f).

**Figure 8:**
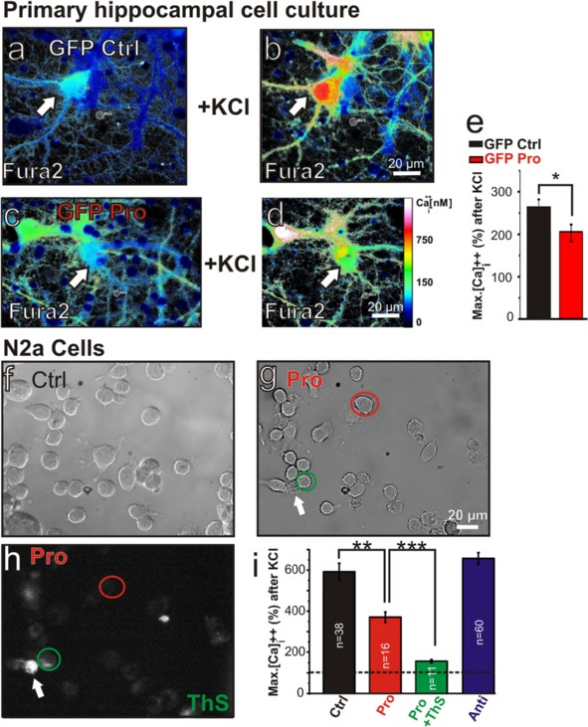
**Expression of pro-aggregant Tau** ^**RDΔ**^ **impairs Ca** ^**++**^ **regulation in primary hippocampal cell culture and N2a cells. (a)** Intracellular Ca^++^ concentrations in primary hippocampal cell culture transfected with GFP only (white arrow) before membrane depolarization by high KCl. Ca^++^ levels are depicted by false color coding according to Fura2 intensity (scale bar in **(d)**). **(b)** Same neuron as in **(a)** after high KCl stimulation. **(c)** Intracellular Ca^++^ levels in a hippocampal neuron expressing pro-aggregant Tau^RDΔ^ and GFP (white arrow). **(d)** Same neuron as in **(c)** after membrane depolarization. **(e)** Quantification of Ca^++^ imaging experiments demonstrating a reduction of calcium influx after high KCl application in pro-aggregant Tau^RDΔ^ expressing cells. Pictures in **(a)**-**(d)** are background subtracted at the indicated location (BG1). **(f)** Bright field image of a N2a control cell culture not expressing pro-aggregant Tau^RDΔ^. **(g)** Bright field image of a N2a cell culture expressing pro-aggregant Tau^RDΔ^. **(h)** *Post-hoc* staining after Ca^++^ imaging experiment of the same cells as in **(g)** by washing in 0.0001% ThioflavineS (ThS) for 5 minutes. ThS enables to distinguish between N2a cells expressing pro-aggregant Tau^RDΔ^ without neurofibrillary tangles (NFT, red circle), N2a cells with NFTs (green circle) and cells with NFT and destroyed membranes (white arrow). **(i)** Quantification of the maximum Ca^++^ influx after KCl stimulation in N2a cells loaded with Fura2AM. Cell populations introduced in **(f)** and **(g)** demonstrating that cells expressing pro-aggregant Tau^RDΔ^ with no ThS positive staining (pro; red column) are already severely impaired but cells with visible tangle load (pro + ThS, green column) are more impaired in Ca^++^ influx. N2a cells expressing anti-aggregant Tau^RDΔPP^ (anti, blue column) do not show reduced calcium influx compared to control cells. Values represent at least three independent experiments. Error bars represent SEM. * p-value < 0.05; ** p-value < 0.01; *** p- value < 0.001.

For comparison we looked at the immediate effect of pro-aggregant Tau^RDΔ^ expression on depolarization induced Ca^++^ influx in rat primary hippocampal neuronal cell cultures. We transiently transfected neurons to express pro-aggregant Tau^RDΔ^ plus GFP, compared with neurons expressing GFP only (Figure 8a-d). There was a pronounced reduction (−60%) of KCl-induced Ca^++^ in Tau^RDΔ^ + GFP expressing neurons (GFP only: 263.4 ± 18.6%; n = 7 and pro-aggregant Tau^RDΔ^ + GFP: 203.4 ± 19.7%; n = 7; p = 0.014, paired T-test; Figure 8e). This confirms that intracellular Tau^RDΔ^ impairs the influx of Ca^++^ after membrane depolarization, both in slices and in primary neurons.

### The reduction of Ca^++^ influx after membrane depolarization is exacerbated with increasing aggregation of pro-aggregant Tau^RDΔ^ in N2a cells

To test if aggregation directly contributes to the observed reduction in Ca^++^ influx after activity induction we made use of an inducible Tet-ON N2a cell line expressing pro-aggregant Tau^RDΔ^ and the reporter gene firefly luciferase (Figure 8f-i) [29]. Induction of pro-aggregant Tau^RDΔ^ expression by doxycycline led to stable expression of the construct, tested via bioluminescence (data not shown). After 4 days of expression ~5% of the cells developed Tau aggregates, which was visualized by Thioflavine-S (ThS) fluorescence (Figure 8g and h). N2a cells expressing pro-aggregant Tau^RDΔ^ displayed a severely reduced KCl induced Ca^++^ influx (−222%) compared to non-induced control cells, even before aggregates became detectable (pro-aggregant Tau^RDΔ^: 370.4 ± 26% of baseline, n = 16; non-induced control cells: 592.07 ± 42% of baseline, n = 38, t-test p < 0.0017; Figure 8i). This effect of pro-aggregant Tau^RDΔ^ was almost doubled (−436%) in N2a cells with ThS detectable tangle load (156.83 ± 8% of baseline; Figure 8h,i). As an additional control we performed experiments with N2a cells expressing anti-aggregant Tau^RDΔPP^, in which evoked Ca^++^ influx was not different from non-induced control cells (657.08 ± 28% of baseline, n = 60; Figure 8i).

We conclude that the pathophysiological effect of Tau is tightly coupled to its propensity for aggregation.

## **Discussion**

Several authors have investigated the influence of pathological Tau on electrophysiological parameters in the hippocampus of transgenic mice. So far these studies were focused on postsynaptic functions in area CA1 [4,6,42,43]. However, in mature neurons Tau is largely restricted to axons, suggesting a presynaptic role [44,45]. In the present study we therefore focused on the physiological and pathophysiological role of axonal Tau and the impact of Tau aggregation on presynaptic morphology and function.

The Tau mutation ΔK280 was first described in a patient with FTDP-17 [46] and later in a case of Alzheimer disease [47]. The mutation affects Tau splicing and is unusual in that it enhances the fraction of 3R-Tau (where repeat R2 is excluded) compared to 4R-Tau, in contrast to many other mutations where 4-R Tau is enhanced. In theory, the mutation of the exon splicer enhancer element in exon 10 would be expected to cause complete exclusion of repeat R2 so that there would be no protein containing the ΔK280 mutation [48], but in the patients a substantial amount of 4R-Tau with the ΔK280 mutation is present in the hippocampus and cortex [47]. Since the mutant protein has a much greater propensity for aggregation compared to wild type Tau [49], and since Tau aggregation is a nucleation-dependent process, nuclei formed by ΔK280 Tau could be elongated even from the pool of normal endogenous Tau and thus poison the entire Tau population [8]. Thus even a small amount of 4R-Tau would be sufficient to initiate pathophysiological events (gain of toxic function). The toxic potential of the mutation is supported by cell and animal models with anti-aggregant Tau (where the propensity for ß-structure is eliminated by insertion of prolines). Mice expressing anti-aggregant Tau^RDΔPP^ show some weak Tau phosphorylation and mislocalization as well as slightly reduced frequency facilitation in the mossy fiber pathway [7,8]. But overall these changes are not sufficient to cause a behavioral phenotype in these mice. Alterations are likely due to an increase in the overall Tau level which may disturb the balance of kinases/phosphatases in the neuron as described previously [50]. In previous studies of the Tau-ΔK280 mutation we had noticed pronounced aggregation, but it was not clear in what pathway the major effect of mutant Tau was located. Here we demonstrate that it is the mossy fiber pathway in the hippocampus that is pronouncedly affected by degeneration. We describe a specific impairment of morphology of Tau-containing presynaptic mossy fiber boutons, which corresponds to an impaired synapse physiology and plasticity. Pronounced pathological phosphorylation, aggregation and missorting occurs in area CA3 in aged mice expressing pro-aggregant Tau^RDΔ^, particularly in the *stratum lucidum*. Tau becomes enriched in mossy fiber giant boutons and reveals the pathognomonic MC-1 epitope in axons and presynapses, arguing for misconformational Tau in the mossy fiber presynapse due to co-aggregation of mutant and endogenous Tau. The mossy fiber boutons displayed a pathological increase of size, observable both in middle- aged mouse brains and in organotypic slices.

The organotypic slice model was further used to analyze the presynaptic structural changes in detail. Giant boutons on granule cell axons were spaced further apart in slices from pro-aggregant Tau^RDΔ^ mice, and at the same time the bouton diameter and 3D surface became larger (~74%). This corresponds to a “condensed presynapse” pathological phenotype when considering the granule cell axon as a whole. We found less but bigger mossy fiber boutons at such axons. Taken together, the excitatory drive of granule cells is decreased due to “condensed presynapses” in pro-aggregant mice, which is reflected in a severely reduced basal synaptic transmission. By contrast, in anti-aggregant animals there was no impairment in basal synaptic transmission or in bouton diameter although endogenous Tau became phosphorylated at AT8 and AT180 site. Looking at the inhibitory site of transmission, we found that filipodia which are known to innervate inhibitory interneurons were slightly increased in number but decreased in length which could account for a compensatory effect. We conclude that aggregation-prone Tau is causal for the reduction in synaptic transmission at the mf-CA3 synapse. Consistent with this, treatment with the aggregation inhibitor bb14 [31] prevented pathological pre- and postsynaptic morphological changes in area CA3 of pro-aggregant Tau^RDΔ^ slices. Data from organotypic slice cultures enabled us to present a comparison of pathological effects on postsynaptic structures (loss of dendritic spines in area CA3: −20%) and the effect of pro-aggregant Tau^RDΔ^ on the presynapse-site (bouton diameter increase: +42%) in parallel. Moreover ultrastructural analysis demonstrated severe reduction of vesicle density in the pathologically modified mossy fiber boutons. Although we cannot rule out the possibility that reduced vesicle density is simply due to an increase in bouton size the finding correlates well with the dramatic effect of pro-aggregant Tau^RDΔ^ on basal synaptic transmission of the mossy fiber pathway (Figure 2a).

Unexpectedly, the depletion of Tau in the TKO mice [25] also led to a pronounced increase in bouton diameter and a concomitant reduction in basal synaptic transmission (Figure 2a), comparable to the phenotype observed in pro-aggregant mice. In pro-aggregant mice, it is likely that an accumulation of aggregating Tau (as visualized by MC1 antibody) in presynaptic terminals leads to an overall increase of the mossy fiber bouton diameter. In contrast, the phenotype observed in TKO mice is more ambiguous. A role of Tau during axon growth and maturation [51] was previously described and primary neurons obtained from the Tau knockout strain showed reduced axon length [25]. This illustrates that Tau reduction can cause structural changes in axons. Why this does not lead to impairment in synaptic plasticity at 13 months remains to be investigated. Nevertheless, the reduction of basal synaptic transmission in the mossy fiber pathway implies a physiological role of Tau as a regulator of neuronal excitability at the DG-CA3 synapse. This might in turn explain how a genetic reduction of Tau could lead to a reduction of seizure susceptibility in hAPP mouse models when crossed with TKO mice [52] and even in non-AD related epilepsy models [53,54]. It has been speculated that the dentate gyrus might act as a “shield” protecting from spreading of epileptiform activity from cortical regions into the hippocampus because of the low intrinsic excitability of dentate granule cells compared with pyramidal cells [55,56]. On the basis of this hypothesis increased seizure susceptibility in hAPP models would be ameliorated by decreased synaptic transmission at the DG-CA3 synapse due to Tau depletion.

Since we did not detect any plasticity impairments in our TKO mice at the mossy fiber-CA3 synapse we conclude that Tau reduction at that synapse could counteract hyperexcitability effects in hAPP mouse models without affecting learning and memory. However a recent study reports plasticity deficits in a different experimental paradigm due to Tau reduction [57], but in contrast to our study deficiencies were found in area CA1.

Subsequently to synaptic transmission we measured mossy fiber short-term plasticity (by paired pulse facilitation and frequency facilitation) and long-term depression (by low frequency stimulation). In contrast to the results on basal synaptic transmission we found that endogenous Tau was indeed not essential for normal mossy fiber short-term plasticity and long-term depression in the mossy fiber pathway. TKO mice were indistinguishable from controls in both parameters in spite of the pathological mossy fiber bouton size increase. However, in middle-aged pro-aggregant mice we observed reduced paired pulse facilitation, frequency facilitation and disrupted LTD expression. This phenotype of impaired mf-plasticity was not present in 2 month-old mice expressing pro-aggregant Tau^RDΔ^ confiming a “phenotype progression” in our mouse model. Paired pulse facilitation is known to depend on presynaptic calcium dynamics [58]. In AD patients a colocalization of A1 receptors with Aß plaques and with tangle bearing pyramidal cells in the hippocampus has been described [59]. Moreover the activation of A1 receptors led to the phosphorylation of Tau and its translocation towards particulate fractions from SH-SY5Y neuroblastoma cells [59]. Given that frequency facilitation is closely correlated with LTD [60] *in vivo* and since proper A1 receptor function is essential for mossy fiber plasticity [20], any modifications of A1 receptor function or transport inhibition might partly contribute to reduced basal synaptic transmission, reduced frequency facilitation and impaired LTD in pro-aggregant animals.

Since the mossy fiber synapse is essential for memory recall, storage and pattern completion [61,62], the described loss of plasticity at this synapse should have deleterious consequences on memory and cognition. This assumption is consistent with previous behavioral observations [7,8] and should therefore be extended in future studies to mossy fiber specific pattern-separation behavioral tests [63].

The major and general cellular prerequisite for bidirectional plasticity is an intact Ca^++^ homeostasis and activity-dependent Ca^++^ influx [64-66]. In particular, at the mf synapse an activity-dependent rise in Ca^++^ levels is necessary for proper LTD expression [23]. We detected a strong reduction of Ca^++^ influx after membrane depolarization at the mossy fiber presynapse, which argues that this is the reason for the pathophysiological effect of Tau^RDΔ^ on presynaptic plasticity. Notably this effect occurs at a time point (DIV 10) where no tangles are present in organotypic slices from pro-aggregant mice, suggesting a soluble, “pre-tangle” form of aggregating Tau that is sufficient for this pathological effect. At an earlier time point (DIV 5) there was only a slight decrease in Ca^++^ influx which underscores that there is a pathological progression in the slice model system. Similarly, two previous studies reported early presynaptic deficits in the enthorinal cortex of Tau transgenic mice [67,68]. Using primary hippocampal cell culture, we found that pro-aggregant Tau^RDΔ^ also led to severe attenuation of Ca^++^ influx in the soma of transiently transfected neurons. This was true even in the absence of Tau aggregates. To investigate if this effect is exacerbated by Tau aggregates, we next used inducible N2a cells expressing the same pro-aggregant Tau^RDΔ^. N2a cells display a prominent Ca^++^ influx mediated by L-type voltage-gated calcium channels (VGCC) as a response to membrane depolarization [69]. Like the primary hippocampal neurons, the N2a cells showed a reduction of Ca^++^ influx due to pro-aggregant Tau^RDΔ^, even before the appearance of ThS-stained Tau aggregates. This effect occurs only with pro-aggregant but not with anti-aggregant Tau^RDΔPP^ and may be explained again by “pre-tangle” oligomeric aggregates which are known to impair cognition in transgenic Tau mice before full-blown tangles appear [9,70,71]. Interestingly, in N2a cells the impairment of Ca^++^ influx was further worsened when ThS-positive Tau aggregates appeared intracellularly. This leads us to propose the activity dependent dysfunction in neuronal Ca^++^ regulation as a cellular patho-mechanism of the observed pre-and postsynaptic tauopathy phenotypes.

## **Conclusions**

In summary we suggest that the pathophysiological effects of pro-aggregant Tau^RDΔ^ on calcium influx and the reduction of synaptic vesicle density causes the impairment of transmission and presynaptic plasticity at the mf-synapse, which will in turn lead to behavioral and cognitive deficits in AD and other tauopathies (Additional file 6: Figure S4).

## Additional files

Additional file 1:
**Supplemental experimental procedures.**
Additional file 2: Table S1. Summary of Tau antibodies. Additional file 3: Figure S1. Mossy fibers are positive for Tauopathy markers detected by immunofluorescence in 13 months old Tau^RDΔ^ mice. (a) Microphotograph of area CA3 showing that there is no detectable PHF-1 immunoreactivity in control littermate mice (Ctrl). (b) In area CA3 of Tau^RDΔ^ expressing mice (Pro) Tau phosphorylation detected by PHF1 immunoreactivity is observed in the mossy fiber pathway (white arrow) in a dotted pattern. (c) Control littermate mice (Ctrl) do not show any AT-8 immunoreactivity in area CA3. (d) Mice expressing Tau^RDΔ^ (Pro) show AT-8 immunoreactivity in area CA3. (e) PHF1 staining displays phosphorylated Tau within the entire lengths of the mossy fibers in pro-aggregant mice (Pro). White arrows indicate weakly stained granule cells in the dentate gyrus. Additional file 4: Figure S2. Expression of exogenous pro- or anti-aggregant Tau^RDΔ^ results in phosphorylation of endogenous mouse Tau and alterations of post- and presynaptic protein levels of the hippocampus. (a) Representative expression of human Tau^RD^ (Mr ~12-14 kDa) and endogenous mouse Tau (mTau, Mr ~45-55 kDa) in pro- and anti-aggregant Tau^RD^ mice compared to control and Tau knockout (TKO) mice. Visualization of non-phosphorylated human and mouse Tau by the pan-Tau antibody K9JA. (b/c) Phosphorylated mouse Tau is only detected in hippocampus homogenates of pro-and anti-aggregant mice by phosphorylation-dependent antibodies AT180 (pT231/pSer235) (b) and AT8 (pSer202/pThr205) (c). (d) Conformational dependent MC-1 immunoreactivity was detected in somata of pyramidal cells in stratum pyramidale of area CA3 and stratum lucidum (asterisk) in pro-aggregant Tau^RDΔ^ mice, but not in control littermates (Ctrl). (e) Representative levels of postsynaptic proteins (PSD95, NMDAR1) and presynaptic proteins (synaptophysin (S’physin), synapsin 1 and piccolo) of CA3 lysates from control, pro- and anti-aggregant and TKO mice. ß-actin serves as loading control. (f) Bars show densiometric analysis of the Western blots (e) for the synaptic proteins (PSD95, NMDAR1, synaptophysin, synapsin 1), all normalized to ß-actin(n = 4). The red (pro-aggregant Tau^RDΔ^) and green (anti-aggregant Tau^RDΔPP^) bars indicate the alteration of the synaptic levels within these mouse strains compared to WT (gray) and TKO (blue) mice. In the pro-aggregant mice the majority of synaptic proteins is reduced (between 60-90% of control level), but the synaptic proteins in the anti-aggregant and TKO mice show similar or higher expression levels than WT mice. Bars represent mean ± SEM. Additional file 5: Figure S3. Axonal and presynaptic localization of Tau and missorting to postsynaptic compartments due to pro-aggregant Tau^RDΔ^ expression. (a) Example of immunohistological detection of endogenous mouse Tau with pan-Tau antibody (K9JA) in *stratum lucidum* of area CA3 b-c of the hippocampus in a control littermate mouse at 13 ± 1 month. Note that strong immunoreactivity (white color) is seen exclusively in the stratum lucidum (s.l., dashed line), where mossy fiber axonal fibers from the dentate gyrus granule cells innervate apical dendrites of CA3 pyramidal cells. Cell somata and dendrites of pyramidal cells are not Tau immunoreactive (note the black "shadows" of non- immunoreactive cell bodies (green arrows) and apical dendrites; s.p. = stratum pyramidale; s.r.= stratum radiatum) (b) Immunoreactivity against endogenous and exogenous Tau in area CA3 of the hippocampus of pro-aggregant (Pro) Tau mice. Tau gets missorted into the somato-dendritic compartment. Strong immunoreactivity (white color) is seen in cell bodies of s.p. in axonal structures in s.l. and in s.r. Arrows point at a pyramidal nucleus (green), an apical dendrite (orange), and mossy fiber bundles in s.l. of area CA3 b-c (dashed line). (c) Control littermate slice (Ctrl) from 13 month old mice stained with a pan-Tau antibody. A region in *stratum lucidum* was magnified in order to show Tau containing mossy fiber presynapse (bouton; highlighted by white dotted circle). (d) DiI labeling (red) of the same region showing the morphology of a “giant” mossy fiber bouton (white dotted circl*e).* (e) Merged picture from (c) and (d) pointing at a “giant” mossy fiber bouton containing Tau (white arrow). (f) K9JA immunoreactivity in the region of the hilus (CA3c) of a pro-aggregant animal (Pro) at the same age as in (c) showing Tau in a "giant" bouton (white dotted circle). Mislocalized, dendritic Tau is indicated by a white arrow. (g) DiI labeling of the same region as in (f) is showing a bouton (white dotted circle) and a neighboring dendrite (white arrow). (h) Merge of picture (f) and (g) showing co-localization of DiI labeling and Tau immunoreactivity. Additional file 6: Figure S4. Schematic illustration of the chain of events leading to synaptic plasticity breakdown due to the aggregation of Tau. In a first step Tau aggregation and phosphorylation lead to a reduced intracellular calcium influx after activity induction. This immediate effect on cellular calcium dynamics is hypothesized to lead to pre-and postsynaptic structural and functional changes. On the presynaptic side aggregation-prone Tau leads to fewer but larger boutons per axon, a net loss of presynaptic markers and to a reduced number of synaptic vesicles finally resulting in a decrease of basal synaptic transmission and short-term plasticity. On the postsynaptic side the dendritic spine density and the important scaffold protein PSD95 are reduced. This - together with presynaptic deficits - is responsible for the deleterious effect of Tau aggregation on long term depression (LTD) of the mossy fiber-CA3 synapse.

## Abbreviations

AD: Alzheimer disease
anti-aggregant Tau^RDΔPP^ = Tau^RD^ΔK280PP: Tau repeat domain with mutation ΔK280 and two additional prolines
CA: Cornu ammonis
Ca^++^: Calcium
DG: Dentate gyrus
FTDP-17: Frontotemporal dementia with parkinsonism linked to chromosome-17
LFS: Low frequency stimulation
LTD: Long term depression
Mf: Mossy fiber
pro-aggregant Tau^RDΔ^ = Tau^RD^ΔK280: Tau repeat domain with mutation ΔK280
TKO: Tau knockout strain
