# Supplementary material for: Pro-aggregant Tau impairs mossy fiber plasticity due to structural changes and Ca++ dysregulation
Source: Acta Neuropathol Commun. 2015 Apr 3;3:23. doi: 10.1186/s40478-015-0193-3 (PMC4384391; doi:10.1186/s40478-015-0193-3)
Supplement: Additional file 1: — Supplemental experimental procedures. [file 40478_2015_193_MOESM1_ESM.pdf]

Supplemental Experimental Procedures

Combined biolistic DiI labeling and immunohistochemistry from acute hippocampal slices (Fig. S3 c-h)

To visualize synaptic structures in acute horizontal slices we used the membrane labeling dye DiI (1,10-dioctadecyl-3,3,30,30-tetramethylindocarbocyanine perchlorate; Invitrogen, Carlsbad, CA, USA) applied by a PSD-1000 / He Particle Delivery System (Bio-Rad Laboratories GmbH). Living, acute hippocampal slices were prepared (see above) with a slice thickness of 150 µm. 2 mg DiI was diluted in 20 µl DMSO. In parallel 0.75mg gold particles (1.6 µm in diameter) were diluted in 250 µl ddH2O, these solutions were mixed and sonicated at 4^0C for 20 min. A single droplet (20 µl) of this solution was pipetted under constant vortexing and placed in the middle of a gene-gun micro-carrier. Acute hippocampal slices were placed in a petri dish and placed at the bottom of the gene gun. Slices were "shot" with 650 psi at a distance of 5 cm; they were immediately transferred to 4% PFA and fixed overnight at 4^0 C. The next day slices were washed 3 times with PBS and then for 30 min in 10 % normal goat serum. As first antibody we used polyclonal (rabbit anti-mouse) K9JA antibody against pan-Tau at a dilution of 1:1000 at 4^0C overnight. After washing 3 times with PBS we subsequently incubated with 1:1000 goat-anti-rabbit secondary (Jackson Immunoresearch) antibody-Cy2 for at least 2 h at RT. After a final washing step slices were mounted onto glass carriers. Since slices were mechanically stressed due to ballistic gold particle bombardment the first antibody was able to penetrate perforated cellular membranes in a fraction of slices without permeabilization.

Immunohistochemistry

Brains from adult 13 month old mice were fixated for 48 h in 4 % PFA at 4^0 C and horizontal, hippocampal free floating sections with a thickness of 80 µm were prepared with a vibratome (Leica VT 1200S, Wetzlar, Germany). Sections were rinsed with 0.4% TritonX in 5 % horse serum for 1 h at RT. After washing with PBS first antibodies (PHF1 (pSer396/pSer404, Dr. P. Davies): 1:250 and AT8 (pSer202/pThr205, 1:500, Thermo Scientific) were incubated at 4°C over night. Secondary antibodies (donkey anti-mouse Alexa 488, Dianova) were incubated (1:100) for 2 h at RT.

Immunoblot analysis (Fig. S2)

Western blots were carried out as described (Mocanu et al., 2008). CA3 region was dissected and 3 - 40 µg of total protein were loaded for the detection with following antibodies: pan-Tau antibody K9JA (1:20000, Dako A-0024), phospho-Tau antibodies AT180 (pThr231/ pSer235, 1:500, Pierce) and antibodies against synaptic proteins: synaptophysin (1:20000, Sigma), synapsin (1:2500, Millipore), PSD95 (1:2000, Dianova) and NMDAR1 (1:500, Millipore). Blots were normalized by the concentration of ß-actin (1:20000, Sigma), developed using the ECL Plus detection system (GE Healthcare) and analyzed by densitometry (LAS 3000, AIDA software, Raytest).
