# Supplementary material for: Pro-aggregant Tau impairs mossy fiber plasticity due to structural changes and Ca++ dysregulation
Source: Acta Neuropathol Commun. 2015 Apr 3;3:23. doi: 10.1186/s40478-015-0193-3 (PMC4384391; doi:10.1186/s40478-015-0193-3)
Supplement: Additional file 2: Table S1. — Summary of Tau antibodies. [file 40478_2015_193_MOESM2_ESM.pdf]

Supplemental Table 1

Table S1: Summary of Tau antibodies

Antibody	Reactivity
K9JA	Human and mouse Tau
PHF1	Tau phosphorylated at Ser396/Ser404
AT8	Tau phosphorylated at Ser202/Thr205
12E8	Tau phosphorylated at S262/S356
MC1	Conformational changes of Tau
AT180	Tau phosphorylated at Thr231/ Ser235
