# Supplementary figures and images for: Pro-aggregant Tau impairs mossy fiber plasticity due to structural changes and Ca++ dysregulation
Source: Acta Neuropathol Commun. 2015 Apr 3;3:23. doi: 10.1186/s40478-015-0193-3 (PMC4384391; doi:10.1186/s40478-015-0193-3)

## Supplemental Figure S1

mf-pathology markers (13 month old mice)

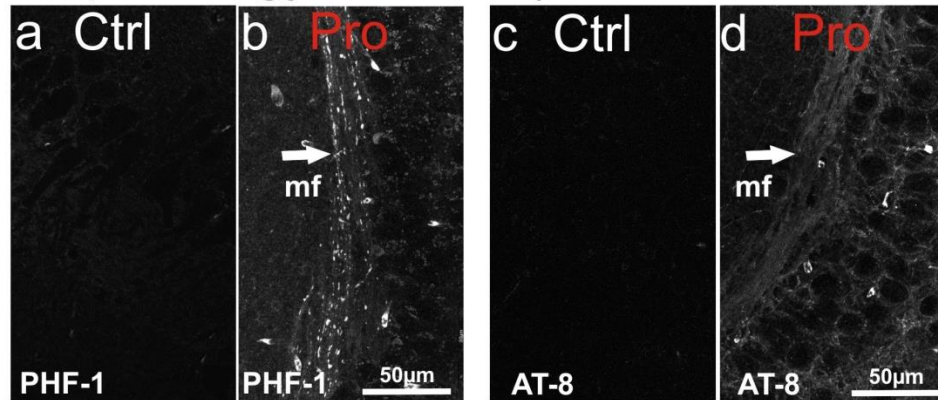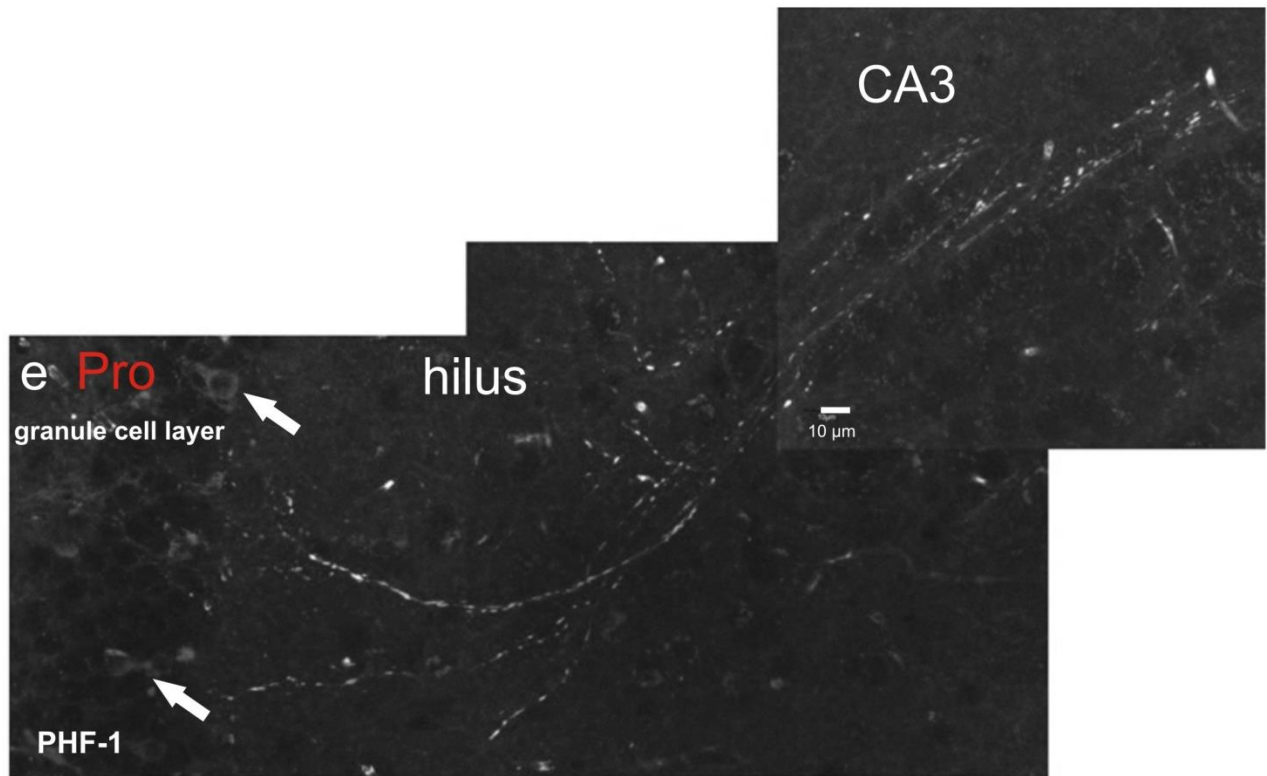

Supplement: Additional file 3: Figure S1. — Mossy fibers are positive for Tauopathy markers detected by immunofluorescence in 13 months old TauRDΔ mice. (a) Microphotograph of area CA3 showing that there is no detectable PHF-1 immunoreactivity in control littermate mice (Ctrl). (b) In area CA3 of TauRDΔ expressing mice (Pro) Tau phosphorylation detected by PHF1 immunoreactivity is observed in the mossy fiber pathway (white arrow) in a dotted pattern. (c) Control littermate mice (Ctrl) do not show any AT-8 immunoreactivity in area CA3. (d) Mice expressing TauRDΔ (Pro) show AT-8 immunoreactivity in area CA3. (e) PHF1 staining displays phosphorylated Tau within the entire lengths of the mossy fibers in pro-aggregant mice (Pro). White arrows indicate weakly stained granule cells in the dentate gyrus. [file 40478_2015_193_MOESM3_ESM.pdf]

# Supplemental Figure S2

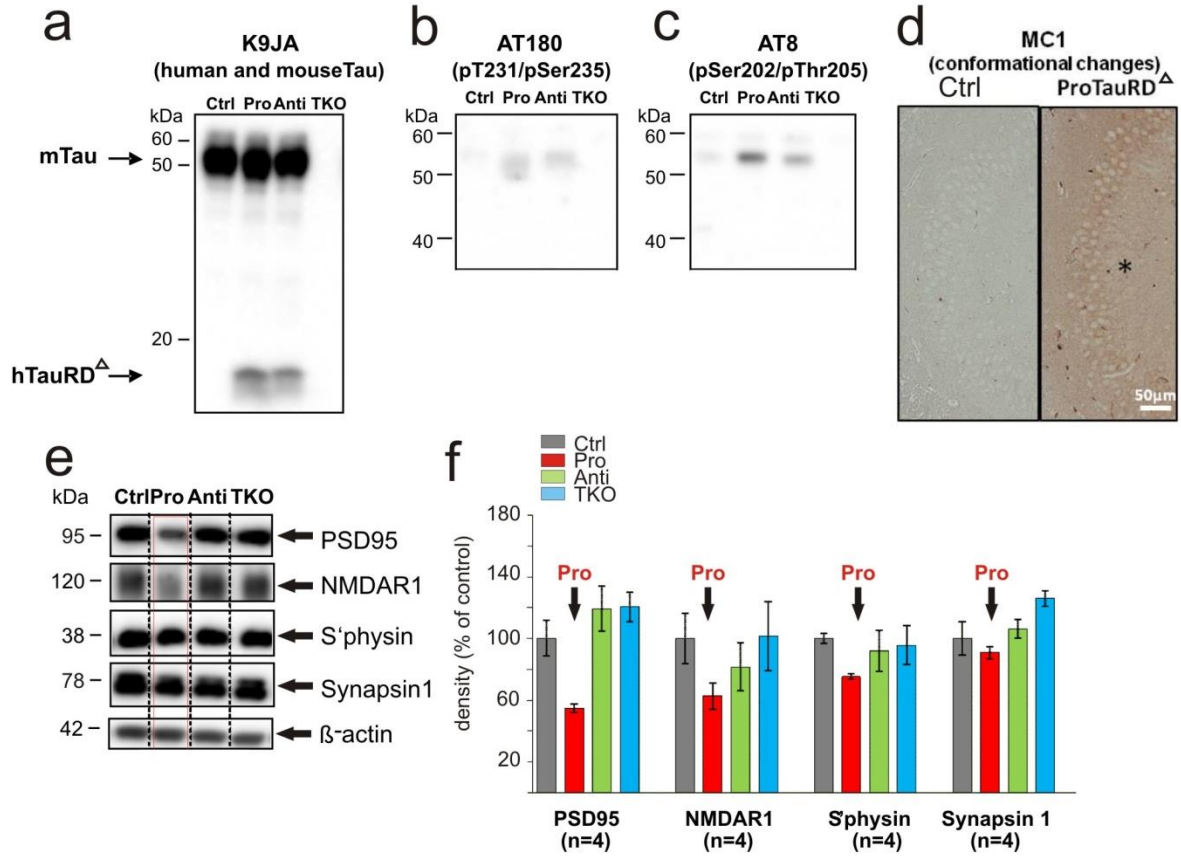

Supplement: Additional file 4: Figure S2. — Expression of exogenous pro- or anti-aggregant TauRDΔ results in phosphorylation of endogenous mouse Tau and alterations of post- and presynaptic protein levels of the hippocampus. (a) Representative expression of human TauRD (Mr ~12-14 kDa) and endogenous mouse Tau (mTau, Mr ~45-55 kDa) in pro- and anti-aggregant TauRD mice compared to control and Tau knockout (TKO) mice. Visualization of non-phosphorylated human and mouse Tau by the pan-Tau antibody K9JA. (b/c) Phosphorylated mouse Tau is only detected in hippocampus homogenates of pro-and anti-aggregant mice by phosphorylation-dependent antibodies AT180 (pT231/pSer235) (b) and AT8 (pSer202/pThr205) (c). (d) Conformational dependent MC-1 immunoreactivity was detected in somata of pyramidal cells in stratum pyramidale of area CA3 and stratum lucidum (asterisk) in pro-aggregant TauRDΔ mice, but not in control littermates (Ctrl). (e) Representative levels of postsynaptic proteins (PSD95, NMDAR1) and presynaptic proteins (synaptophysin (S’physin), synapsin 1 and piccolo) of CA3 lysates from control, pro- and anti-aggregant and TKO mice. ß-actin serves as loading control. (f) Bars show densiometric analysis of the Western blots (e) for the synaptic proteins (PSD95, NMDAR1, synaptophysin, synapsin 1), all normalized to ß-actin(n = 4). The red (pro-aggregant TauRDΔ) and green (anti-aggregant TauRDΔPP) bars indicate the alteration of the synaptic levels within these mouse strains compared to WT (gray) and TKO (blue) mice. In the pro-aggregant mice the majority of synaptic proteins is reduced (between 60-90% of control level), but the synaptic proteins in the anti-aggregant and TKO mice show similar or higher expression levels than WT mice. Bars represent mean ± SEM. [file 40478_2015_193_MOESM4_ESM.pdf]

## Supplemental Figure S3

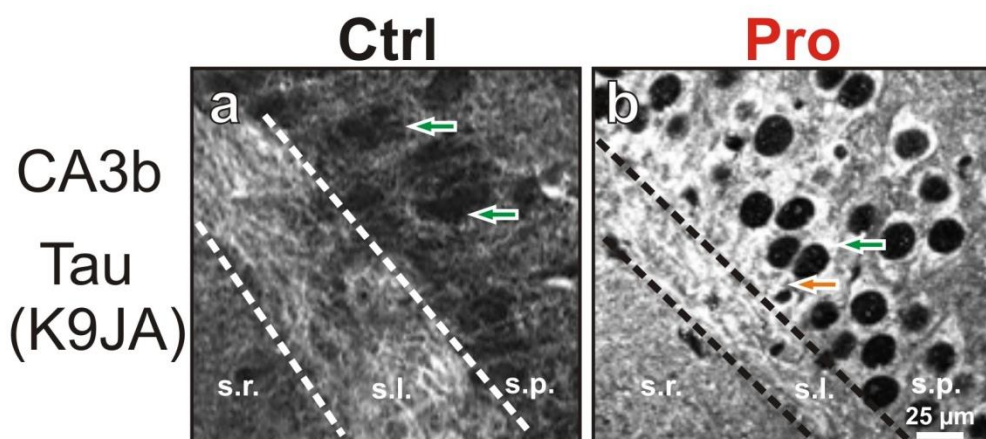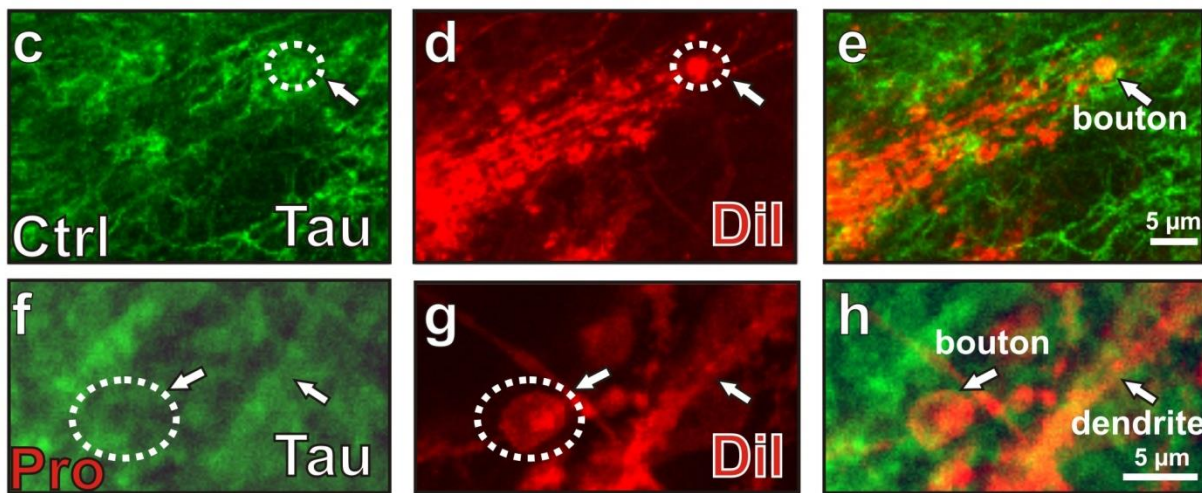

Supplement: Additional file 5: Figure S3. — Axonal and presynaptic localization of Tau and missorting to postsynaptic compartments due to pro-aggregant TauRDΔ expression. (a) Example of immunohistological detection of endogenous mouse Tau with pan-Tau antibody (K9JA) in stratum lucidum of area CA3 b-c of the hippocampus in a control littermate mouse at 13 ± 1 month. Note that strong immunoreactivity (white color) is seen exclusively in the stratum lucidum (s.l., dashed line), where mossy fiber axonal fibers from the dentate gyrus granule cells innervate apical dendrites of CA3 pyramidal cells. Cell somata and dendrites of pyramidal cells are not Tau immunoreactive (note the black "shadows" of non- immunoreactive cell bodies (green arrows) and apical dendrites; s.p. = stratum pyramidale; s.r.= stratum radiatum) (b) Immunoreactivity against endogenous and exogenous Tau in area CA3 of the hippocampus of pro-aggregant (Pro) Tau mice. Tau gets missorted into the somato-dendritic compartment. Strong immunoreactivity (white color) is seen in cell bodies of s.p. in axonal structures in s.l. and in s.r. Arrows point at a pyramidal nucleus (green), an apical dendrite (orange), and mossy fiber bundles in s.l. of area CA3 b-c (dashed line). (c) Control littermate slice (Ctrl) from 13 month old mice stained with a pan-Tau antibody. A region in stratum lucidum was magnified in order to show Tau containing mossy fiber presynapse (bouton; highlighted by white dotted circle). (d) DiI labeling (red) of the same region showing the morphology of a “giant” mossy fiber bouton (white dotted circle). (e) Merged picture from (c) and (d) pointing at a “giant” mossy fiber bouton containing Tau (white arrow). (f) K9JA immunoreactivity in the region of the hilus (CA3c) of a pro-aggregant animal (Pro) at the same age as in (c) showing Tau in a "giant" bouton (white dotted circle). Mislocalized, dendritic Tau is indicated by a white arrow. (g) DiI labeling of the same region as in (f) is showing a bouton (white dotted circle) and a neighbo [file 40478_2015_193_MOESM5_ESM.pdf]

## Supplemental Figure S4

### Tau phosphorylation/aggregation

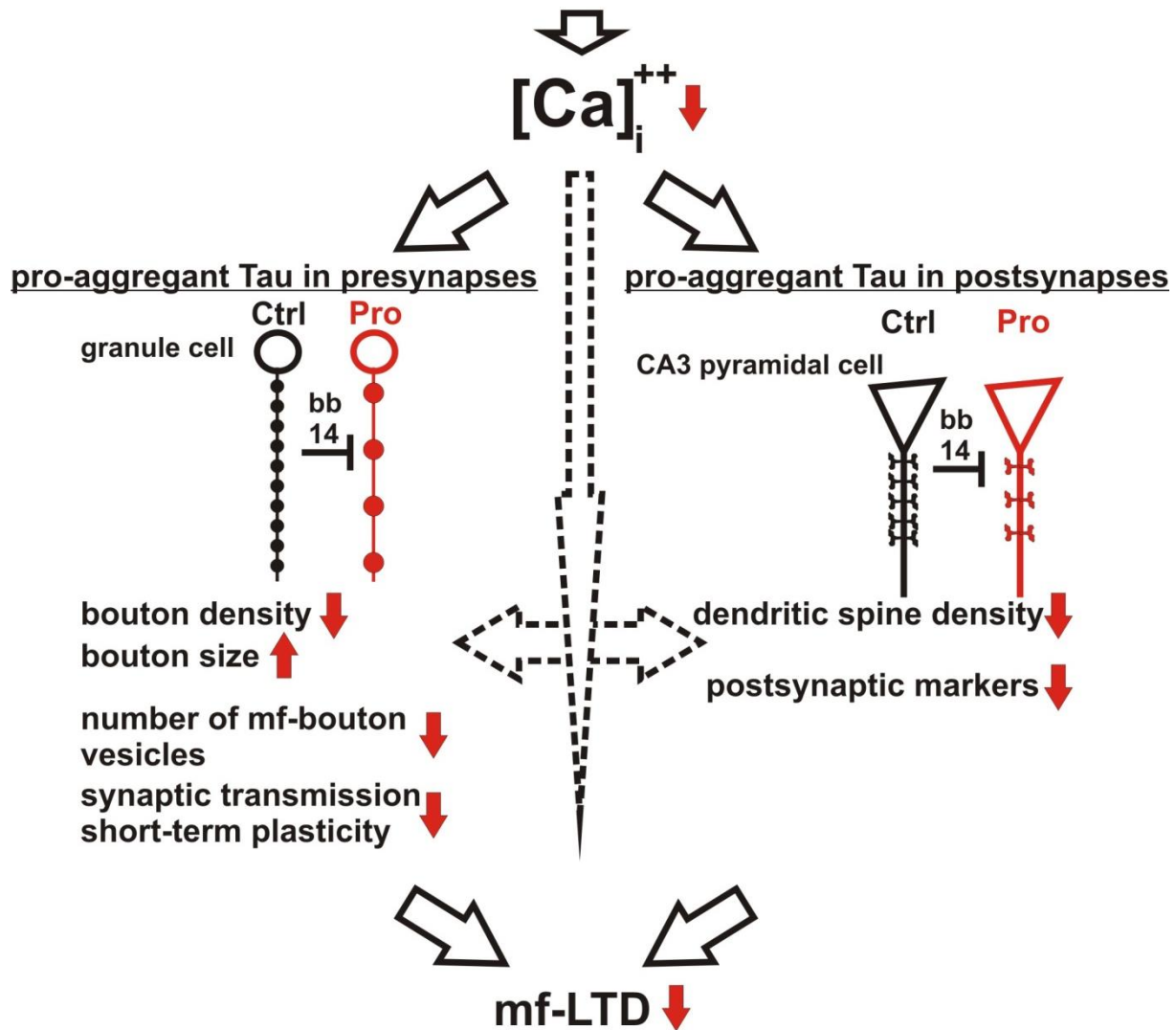

Supplement: Additional file 6: Figure S4. — Schematic illustration of the chain of events leading to synaptic plasticity breakdown due to the aggregation of Tau. In a first step Tau aggregation and phosphorylation lead to a reduced intracellular calcium influx after activity induction. This immediate effect on cellular calcium dynamics is hypothesized to lead to pre-and postsynaptic structural and functional changes. On the presynaptic side aggregation-prone Tau leads to fewer but larger boutons per axon, a net loss of presynaptic markers and to a reduced number of synaptic vesicles finally resulting in a decrease of basal synaptic transmission and short-term plasticity. On the postsynaptic side the dendritic spine density and the important scaffold protein PSD95 are reduced. This - together with presynaptic deficits - is responsible for the deleterious effect of Tau aggregation on long term depression (LTD) of the mossy fiber-CA3 synapse. [file 40478_2015_193_MOESM6_ESM.pdf]
